# Control of long-distance cell-to-cell communication and autophagosome transfer in squamous cell carcinoma via tunneling nanotubes

**DOI:** 10.18632/oncotarget.15467

**Published:** 2017-02-18

**Authors:** Inés Sáenz-de-Santa-María, Cristóbal Bernardo-Castiñeira, Eduardo Enciso, Inmaculada García-Moreno, Jose Luis Chiara, Carlos Suarez, María-Dolores Chiara

**Affiliations:** ^1^ Servicio de Otorrinolaringología, Hospital Universitario Central de Asturias, Instituto Universitario de Oncología del Principado de Asturias, CIBERONC, Universidad de Oviedo, Oviedo, Spain; ^2^ Facultad de Ciencias Químicas, Universidad Complutense de Madrid, Madrid, Spain; ^3^ Instituto Química-Física “Rocasolano”, IQFR-CSIC, Madrid, Spain; ^4^ Instituto de Química Orgánica General, IQOG-CSIC, Madrid, Spain

**Keywords:** tunneling nanotubes, cell communication, FAK, MMP-2, squamous cell carcinomas

## Abstract

Tunneling nanotubes (TnTs) are thin channels that temporally connect nearby cells allowing the cell-to-cell trafficking of biomolecules and organelles. The presence or absence of TnTs in human neoplasms and the mechanisms of TnT assembly remains largely unexplored. In this study, we have identified TnTs in tumor cells derived from squamous cell carcinomas (SCC) cultured under bi-dimensional and tri-dimensional conditions and also in human SCC tissues. Our study demonstrates that TnTs are not specific of epithelial or mesenchymal phenotypes and allow the trafficking of endosomal/lysosomal vesicles, mitochondria, and autophagosomes between both types of cells. We have identified focal adhesion kinase (FAK) as a key molecule required for TnT assembly via a mechanism involving the MMP-2 metalloprotease. We have also found that the FAK inhibitor PF-562271, which is currently in clinical development for cancer treatment, impairs TnT formation. Finally, FAK-deficient cells transfer lysosomes/autophagosomes to FAK-proficient cells via TnTs which may represent a novel mechanism to adapt to the stress elicited by impaired FAK signaling. Collectively, our results strongly suggest a link between FAK, MMP-2, and TnT, and unveil new vulnerabilities that can be exploited to efficiently eradicate cancer cells.

## INTRODUCTION

Cell-to-cell communication is a mechanism for the transit of information essential for coordination of cellular events in multicellular systems. These include direct cell-cell contacts and cellular interactions via the endocrine, nervous and immune systems. Over the years, it has become clear that disorders of these intercellular communications are important in the pathogenesis of some diseases, in particular cancer. The most widely studied mechanisms of intercellular communication among cancer cells involve soluble factors [1], exosomes [2], and tight, adherence or gap junctions [3]. However, the mechanisms of direct cell-to-cell communication among distant cells in the spatially constrained three-dimensional architecture of invasive tumors remain largely unexplored.

Tunneling nanotubes (TnT), recently discovered in mammalian cells, have been recognized as a novel system of direct cell-to-cell interaction [4]. TnTs are thin tube structures that connect distant cells forming membrane bridges. They have been proved to be a viable mechanism of exchange of biomolecules, pathogens, and organelle between connected cells [5–12]. These intercellular bridges are filled with cytoskeletal filaments, like actin, and may also contain microtubules and motor proteins [10, 13–15]. They were first described in rat pheochromocytoma PC12 cells [14]. Since then, TnTs formation has been observed in a wide range of non-transformed cells *in vitro* [16, 17]. Recent reports have also demonstrated the existence of TnTs in several cancer cell types [13, 18–20].

The molecular basis of TnTs formation is still not fully understood. Several reports suggested that polymerization of actin is required for TnT assembly via the Akt/PI3K/mTOR signaling pathway [21, 22]. Actin dynamics are also regulated by signaling networks downstream of integrins localized at focal adhesion sites [23]. The role of those actin-related signaling networks on TnT dynamics is, thus far, unknown. Given the close relationship of focal adhesion sites with cell-cell contacts, extracellular matrix (ECM), microtubule and actin regulation, it is plausible that these structures play an important role in TnT assembly.

In the present report, we disclose that cell-to-cell communication through TnTs is a common feature of cancer cell lines derived head and neck squamous cell carcinomas (SCC) irrespective of their epithelial or mesenchymal phenotype. Importantly, we found that TnTs allow the trafficking of endosomal/lysosomal vesicles, autophagosomes and mitochondria between both types of cells. We also show that inhibition of Focal adhesion kinase (FAK) signaling dramatically reduced TnT formation and that this phenotype can be reversed by overexpression of the MMP-2 metalloprotease. These data support the conclusion that FAK regulates TnT assembly by promoting MMP-2 production.

## RESULTS

### Long cellular projections identified in SCC-derived cell lines are morphologically and functionally similar to tunneling nanotubes

Two types of cellular long projections were observed and morphologically characterized in cell lines derived from head and neck SCC (Supplementary Data and Supplementary Figure 1). One of them, established cell-cell contacts and were morphologically similar to the so-called tubular nanotubes (TnT).

**Figure 1:**
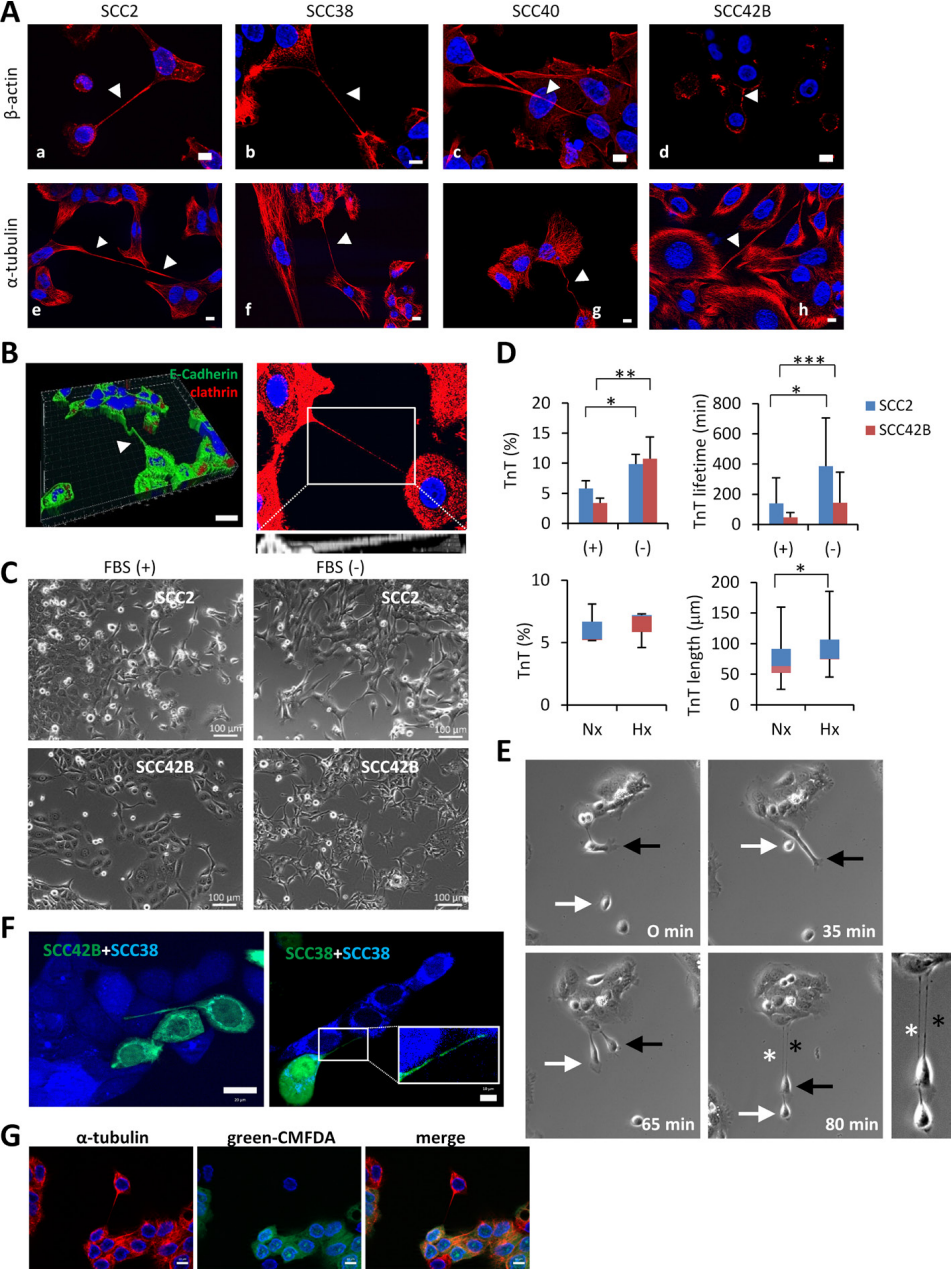
Similarities of long cell projections in SCC-derived cells with TnTs (**A**) Representative images of staining for β-actin and α-tubulin in the indicated cell lines (white arrows points to TnT projections). Image in c is a 76 μm Z-projection of 17 optical sections showing a TnT that crosses above the nuclei of an adjacent intermediate cell thus suggesting that TnTs are not attached to the culture plate surface but hover over it. Scale bars, 10 μm. (**B**) Left, three-dimensional reconstruction of Z-stacked images using Imaris software showing a TnT that hovers over the culture plate surface. Cells were immunostained with the indicated antibodies. The resulting image represents a stack of 9 sections (Z step of 2.8 μm) with a total physical length of 25.2 μm. Scale bar, 30 μm. Right, 17.3 μm Z-projection of 9 optical sections of SCC2 cells immunostained with β-catenin. The projection allows visualization of the whole length of the TnT which could not be visualized in a single z dimension. At the bottom is shown an xz projection of inset area, demonstrating that the TNT run above the surface of the substrate. (**C**) Representative images of the indicated SCC cells cultured in the presence (+) or absence (−) of FBS for 24 hours. Scale bars, 100 μm. (**D**) Up, quantification of percentages and lifetime of TnTs in cells treated as indicated in panel C. Down, quantification of percentages and length of TnTs in cell incubated under normoxic (21% O_2_, 24 h) or hypoxic (1% O_2_, 24 h) conditions. Cells were pictured by using a bright field microscope and an 8×8 tile was used for TnTs identification and counting. Data are presented as mean ± standard deviation from 2 individual experiments and 800 cells analyzed in each.* indicates *P* < 0.05, ** indicates *P* < 0.005, *** indicate *P* < 0.0005. (**E**) Representative images of SCC38 cells from time-lapse movies at the indicated time points. White and black arrows point to two different cells involved in the assembly of two TnT projections which are denoted by white and black asterisks, respectively. A magnified picture of the two TnTs formed at 80 min is shown at the right. (**F**) Fluorescence images of co-cultures of SCC42B cells, labeled with CellTracker green CMFDA, and SCC38 cells, labeled with CellTracker blue CMAC (left) or SCC38 cells independently labeled with either green CMFDA or blue CMAC (right). Inset (right picture) shows a magnified image of the TnT. (**G**) Immunofluorescence analysis of α-tubulin in co-culture of SCC38 cells labeled and not labeled with green CMFDA.

In depth analysis of these structures and the TnTs assembled in PC12 cells, which represent the cellular system where TnTs were first identified [14], revealed that the TnTs formed in SCC cells were 1.8-2.3-fold thicker, more durable (1.6-fold), and 2-5-fold larger in length than TnTs of PC12 cells (Supplementary Figure 2). According to the literature, the TnTs of PC12 cells contain only F-actin whereas UV-damaged cells form a different type of TnTs which have increased diameter and contain microtubule in addition to F-actin [24]. However, in our culture conditions, we found that all TnTs of PC12 cells contained both, microtubules and F-actin (Supplementary Figure 2). Similarly, immunostainings of α-tubulin and β-actin showed that not only F-actin but also microtubules were localized inside the cell projections of SCC cells (Figure 1A). As previously described for TnTs [14], cell projections of SCC cells also hovered freely in culture medium as shown in Figure 1Ac which represents a Z-projection of 17 optical sections showing a TnT that crosses above the nuclei of an adjacent intermediate cell. This is also demonstrated by three-dimensional reconstructions of Z-stacked images or XZ projection of cells that highlights TnTs running above the surface of the substrate (Figure 1B).

The development of TnTs has been shown to be a property of cells under stress [21], a condition that can be induced in culture by the withdrawal of serum. Accordingly, TnTs were induced by serum deprivation increasing both in number and in lifetime (Figure 1C and 1D). When subjected to other stressful condition such as hypoxia (1% O_2_ for 24 hours), cell projections significantly increased their length but they did not increase in number suggesting that not only stressful conditions but also extracellular signals control the assembly of TnTs.

Time-lapse video microscopy revealed that detachment of connected cells is the primary mechanism responsible for TnT biogenesis in SCC cells (Figure 1E, Supplementary Figure 3 and Supplementary Videos 1–3). Accordingly, SCC cells formed TnTs by retaining a progressively thinner thread of membrane upon cell dislodgement (100% of > 1000 formation events visualized). This seems to exclude the possibility that TnTs are formed between dividing cells or that are the result of the convergence of protruding filopodia from neighboring cells. Nevertheless, the fact that we have not been able to visualize formation of TnTs via filopodium protrusions does not strictly exclude the very remote possibility that this mode of assembly can take place between some cells. Because the initial characterization of TnTs were performed in fixed-cells which are caught at specific stages of TnT formation, the measurements of tube-diameters (Supplementary Figure 1) could have been overestimated. Analysis under time-lapse videomicroscopy revealed that the minimum diameters of TnTs are under 1 μm in all SCC cell lines. Thus, although other terms, such as membrane microtubes, have been used to describe these types of tubular structures, the observed nano range dimension of their width at the final stages of formation lead us to maintain the terminology of TnTs for the membrane tubes formed in SCC cells.

To unravel whether TnTs belong to a single cell or to both connected cells, co-culture of two populations of SCC cells labeled with different cell-permeable fluorescent dyes was performed. Figure 1F shows co-cultures of SCC42B cells labelled with CellTracker green CMFDA and SCC38 labelled with CellTracker blue CMAC (left panel) and co-cultures of two populations of SCC38 cells independently labelled with either blue or green dyes (right panel). The data revealed that TnTs are mostly stained by a single color thus demonstrating that they are formed from a single cell (*n* = 210 TnTs analyzed: 1 mixed colored TnT, 209 single colored TnT). Immunostaining with α-tubulin in co-cultures of mixed green+non-fluorescently labeled cells shows that cell connections via TnTs are similar to those identified previously (Figure 1G). These data also demonstrate that the structures that we identified as TnTs do not correspond to any tubular connection formed between sister cells after cell division.

### In-depth microscopy analysis of TnTs

**Figure 2:**
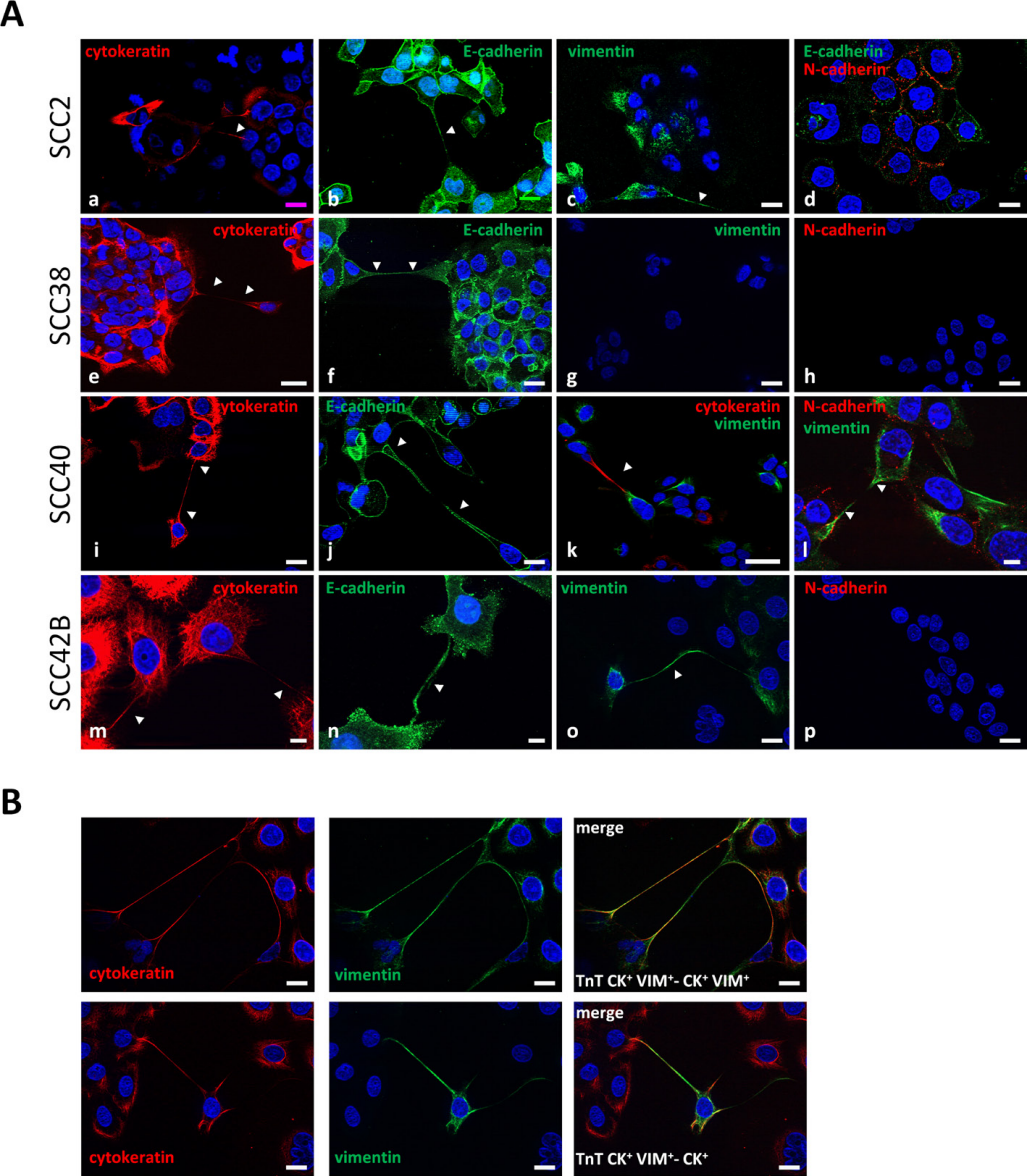
TnTs establish connections between mesenchymal and epithelial cancer cells (**A**) Representative images of staining for cytokeratin, E-cadherin, vimentin, and N-cadherin in the indicated cell lines. The image of E-cadherin in SCC2 and SCC42B cells are a 2.8 μm and 17.3 μm Z-projection, respectively, of 9 optical sections which allows visualization of the whole length of a TnT hovering over the culture surface given that whole TnT could not be visualized in a single z dimension. Arrows indicate CK+-CK+ (a, e, i, m), E+-E+ (b, f, j, n), VIM+-VIM+ (c, l, o), and CK+-VIM+ (k) TnTs. Scale bars, 10 μm (l-n), 20 μm (a-j, o, p), 50 μm (k). (**B**) Representative images of co-staining for cytokeratin and vimentin of SCC42B cells showing CK+VIM+-TnTs connecting a CK+-cell or a CK+VIM+-cell as indicated. Scale bars 18 μm.

The presence of major cytoskeletal components in TnTs was analyzed by immunocytochemistry (Figure 2). SCC38 cells were shown to be a homogeneous cell line with an epithelial-like phenotype as demonstrated by the expression of epithelial (cytokeratin -CK- and E-cadherin -E-), but not mesenchyme (vimentin -VIM- and N-cadherin -N-) cell markers and, thus, TnTs of SCC38 cells were CK^+^E^+^/VIM^−^N^−^. The SCC40 and SCC2 cell lines, in contrast, had mixed cell populations of epithelial (CK^+^E^+^/VIM^−^N^−^) and mesenchymal (VIM^+^N^+^/CK^−^E^−^) phenotypes (SCC2: 23% epithelial cells; SCC40: 46.3% epithelial cells, 320 and 1049 cells counted, respectively). In these cell lines, TnTs emerging from the two cell types were found. The SCC42B cell line had cell populations that were either CK^+^ (55% of cells) or CK^+^VIM^+^ (45% of cells) (200 cells counted) both of them expressing E-cadherin, but not N-cadherin and, thus, TnTs were either CK^+^E^+^/VIM^−^N^−^ or CK^+^VIM^+^E^+^/N^−^. We found that connections through TnTs could be established between cells of the same (CK^+^-cell-to-CK^+^-cell, VIM^+^-cell-to-VIM^+^-cell or CK^+^VIM^+^-cell-to-CK^+^VIM^+^-cell) or different phenotypes (CK^+^-cell-to-VIM^+^-cell, or CK^+^-cell-to-CK^+^VIM^+^-cell), (Figure 2B) thus indicating that TnTs are not a specific feature of an epithelial or mesenchyme cancer cell phenotype.

**Figure 3:**
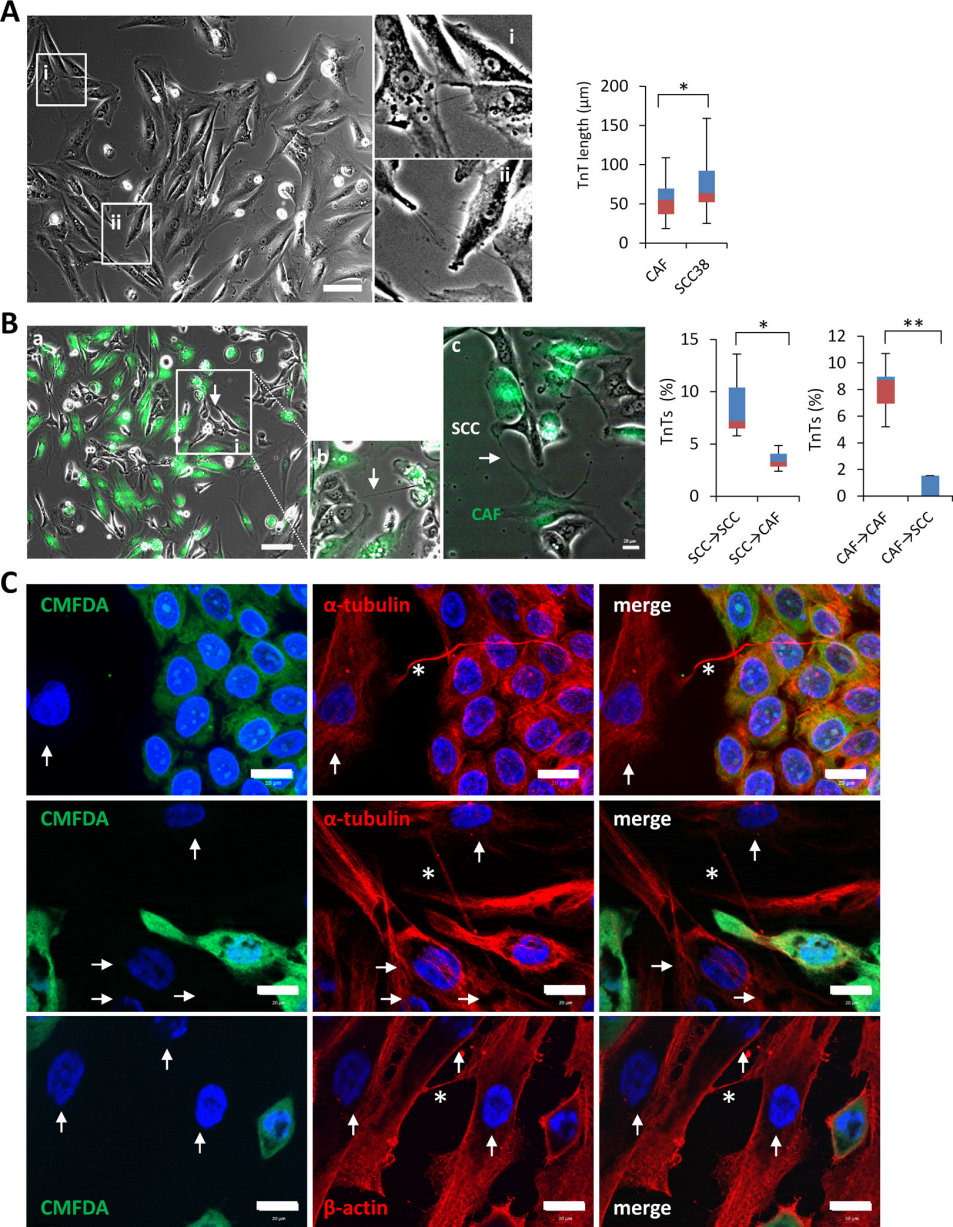
TnTs emanating from SCC cells preferentially connect with other cancer cells rather than to cancer-associated fibroblasts (**A**) Phase contrast image of cancer associated fibroblasts. Insets at the right (i and ii) show magnified images to better visualize the presence of TnTs connecting fibroblasts. Cells were pictured by using a bright field microscope and an 8×8 tile was used for TnTs identification and counting. Graph shows quantification of TnT length in fibroblasts as compared to those of SCC38 cells. Data are presented as mean ± standard deviation from 2 individual experiments and 380 cells analyzed in each. * indicates *P* < 0.05. (**B**) Representative phase contrast plus green fluorescence image of CAFs, labeled with green CMFDA, and SCC cells co-culture. Inset (b) shows a magnified image to better visualize the TnTs connecting a SCC cell with a fibroblast. Image c shows a green TnT that emanates from a CAF and establishes contact with a SCC cell. Cells were pictured by using a bright field plus fluorescence microscope and an 8×8 tile was used for TnTs identification and counting. Graphs show quantification of the percentage of TnTs connecting SCC38-to-SCC38 cells (SCC38→SCC38), SCC38-to-CAFs (SCC38→CAF), CAF-to-CAF (CAF→CAF) or CAF-to-SCC38 cells (CAF→SCC38). Data are presented as mean ± standard deviation from 2 individual experiments and 570 cells analyzed in each. White arrows point to TnTs. * indicates *P* < 0.05, ** indicate *P* < 0.005. (**C**) Representative images of staining for β-actin and α-tubulin in co-cultures of CAFs and SCC38 cells. Adherent SCC38 cells were fluorescently labeled with CellTracker green CMFDA before the addition of un-labeled CAFs. Co-cultured cells were incubated at 37°C for 10–12 hours to allow for CAFs adhesion and TnT formation before immunofluorescence staining. Arrows and asterisks point to CAFs and TnTs, respectively.

We then tested whether TnT formation is a specific feature of SCC cells or whether cell-to-cell contacts through TnTs could also be established between stromal cells such as cancer associated fibroblasts (CAF). As shown in Figure 3A, TnTs were also found in CAFs derived from a human head and neck SCC (Supplementary methods and Supplementary Figure 4). These cell structures were morphologically similar to those emerged from SCC cells with the only difference being that TnTs of CAFs were shorter in length than those of SCC38 cells (Figure 3A) which is the cell line that assemble longer TnTs. Comparison between SCC42B cells and CAFs also revealed a significantly shorter TnT length in CAFs cells than SCC42B [77.27 ± 21.34 μm for SCC42B (*n* = 1200 cells counted) and 56.35 ± 21.45 μm for CAFs, (*n* = 400 cells counted), *p* = 0.023] although differences were of smaller magnitude than that observed with SCC38 cells. We next performed co-cultures of CAFs, which were labeled with CellTracker green CMFDA, and SCC cells to determine whether heterotypic CAF-to-SCC contacts through TnTs were formed. As shown in Figure 3B, although some green TnTs that connect CAFs with SCC38 cells or TnTs that emerge from SCC38 cells and connect with CAFs were found, these were significantly less abundant than the monotypic (i.e, CAF-CAF or SCC38-SCC38) contacts mediated by TnTs. The results were similar when SCC cells, instead of CAFs, were labeled with CellTracker green CMFDA (Figure 3C). Immunofluorescence labeling of β-actin or α-tubulin in these conditions revealed that, as TnTs of SCC cells, those identified in CAFs also contained β-actin and α-tubulin (Figure 3C).

### TnT are formed in three-dimensional cultures and tumor tissues

**Figure 4:**
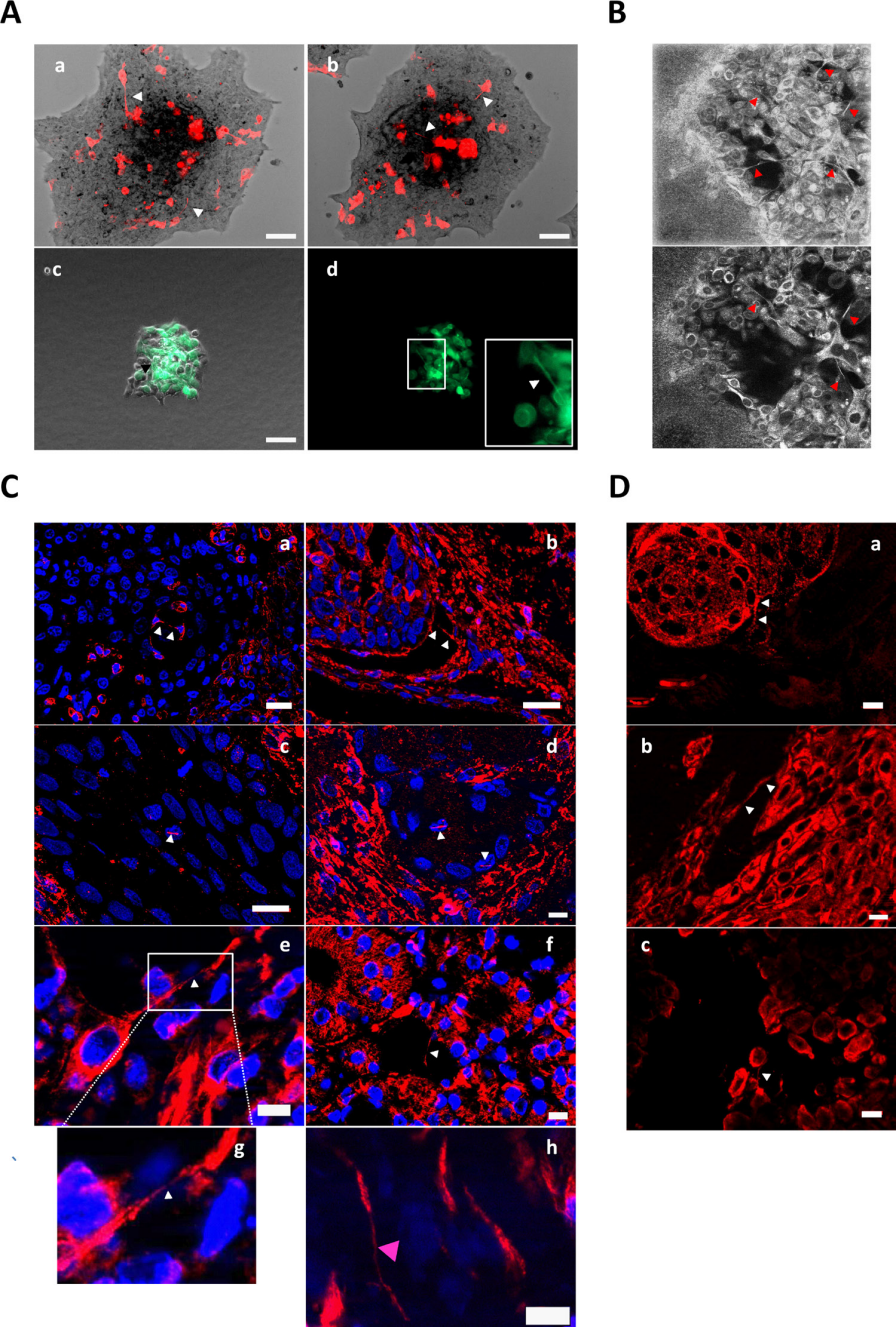
Detection of TnTs in tumor spheres, human SCC tissues and mouse tumor xenograft (**A**) Representative images from time-lapse movies of SCC42B cells embedded as tumor-spheres into collagen matrix and allowed to migrate for 48 h (a and b shows two different tumor-spheres) or 14 h (c and d shows the same tumor-sphere imaged with phase contrast plus green fluorescence -c- or only with green fluorescence -d-). Cells were transiently transfected with LifeAct vector (a, b) or were labeled with CMFDA (green) and, subsequently, mixed (1:1) with non-labeled cells (c, d). Scale bars 100 μm. (**B**) SCC42B cells cultured as tumor-spheres were allowed to migrate for 24 h, and subsequently they were fixed and imaged by confocal reflection microscopy which allows visualizing the collagen matrix. Upper image is a 58 μm Z-projection of 19 optical sections. (**C**) Immunohistofluorescence detection of β-actin (a-d) or α-tubulin (e-h) in human SCC tissue showing TnT-like structures crossing over tumor cell nuclei or connecting independent cancer cells. TnTs of cancer cell are pointed by white arrows. TnT of a tumor stroma cell (h) is denoted by a pink arrow in panel h. Image in panel g shows the magnified image that is indicated in panel e which allows better visualization of the cellular tubular structure crossing over a nuclei located in a subjacent z plane. Scale bars 10 μm. (**D**) Immunohistofluorescence detection of β-actin in primary SCCs developed in nu/nu mice by inoculation of the tumoral cell lines SCC38 (a and b) or SCC42B (c). Arrows denote the TnTs. Scale bars 10 μm.

We next sought to determine whether TnTs are formed in three-dimensional cell culture models, which better mimic the phenotypic and morphological behavior of tumor cells *in vivo* [25]. For these experiments, SCC42B cells were transiently transfected, under low efficiency conditions, with the fluorescent LifeAct vector to be able to visualize fluorescent TnTs emitted by transfected cells over the non-stained background formed by non-transfected cells. Transfected cells were embedded in collagen to form tumor-spheres and were allowed to migrate and invade the artificial ECM. Microscopic video recording revealed that, under these circumstances, cells launched TnTs that established contacts with distant cells passing over other nearby cells (Figure 4Aa-b). This data also shows that, as mentioned above, TnTs seem to mostly emanate from a single cell. Similarly, TnTs could be visualized inside tumor-spheres by confocal reflection microscopy (Figure 4B), and also inside tumor-spheres formed by mixed populations of cells labeled with Celltracker Green CMFDA and non-stained cells (Figure 4Ac-d). Although the three-dimensionality of tissues does not allow assuring whether TnTs are formed *in vivo*, immunohistochemistry of β-actin or α-tubulin in tissues from human head and neck SCCs revealed the presence of actin- and tubulin-containing filamentous structures that cross above cell nuclei or connect independent cells (Figure 4C). We also sought to analyze orthotropic tumor xenograft models of head and neck SCC derived from SCC38 and SCC42B cells (Supplementary methods) that should allow visualization of TnTs over the background of non-stained mouse cells. We were able to detect projections contacting SCC cells but, however, we did not found TnTs that, emerging from α-actin-stained SCC cells, established contact with stromal cells surrounding the tumor niches (Figure 4D). This is in agreement with the *in vitro* experiments that revealed that TnT-mediated heterotypic cell contacts are not frequently formed. Thus, TnTs may serve to establish monotypic cancer cell communications and are not a consequence of two-dimensional culture conditions but are also formed in tumor tissues.

### Organelle trafficking along TnT in SCC-derived cells

**Figure 5:**
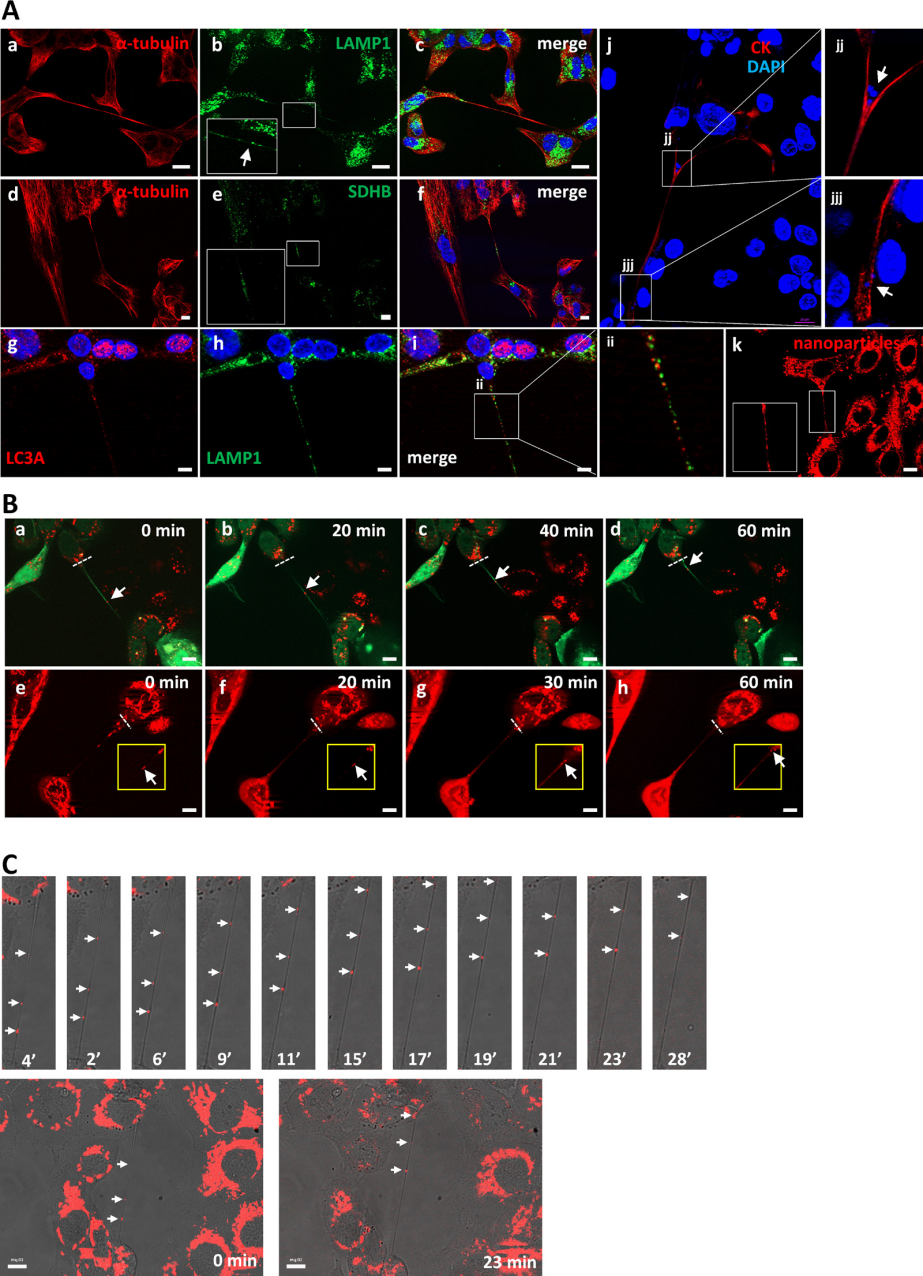
Presence and traffic of organelles inside TnTs (**A**) Representative images of immunostaining for α-tubulin plus LAMP1 in SCC2 cells (a-c), or α-tubulin plus SDHB (d-f), LC3A plus LAMP1 (g-i), or α-tubulin (j) in SCC38 cells. Panel k shows a representative image of SCC2 cells loaded *in vivo* with rhodamine-containing nanoparticles. Insets in b, e, and k show magnified images to better visualize the presence of positive signals for LAMP1, SDHB, and nanoparticles, respectively, inside the TnTs. Scale bars, 20 μm. Panels jj, jjj and ii show magnified images indicated in panels j and i to better visualize the presence of positive signals for DAPI (j, jj) and the localization of LAMP1 and LC3A positive vesicles. (**B**) Representative images from time-lapse movies at the indicated time points. SCC38 cells were labeled *in vivo* with green CMFDA plus lysotracker (a-d) or mitotracker (e-h). Dashed white lines represent the reference position taken for quantification of the rate of organelle movements. Scale bars, 10 μm. (**C**) Representative merged transmitted light and fluorescence images from time-lapse movies at the indicated time points. SCC38 cells were loaded with rhodamine-containing nanoparticles (5.3 × 10^−5^ % of nanoparticles). Directional movement was not observed when using higher concentrations of nanoparticles (5.3 × 10^−3^ %) likely due to the formation of aggregates that could get clogged inside the nanotube. Images at time 0 and 23 min are shown in the bottom to illustrate the presence of the TnT connecting the two cells. White arrows denote nanoparticles.

By immunocytochemical analysis using anti-LAMP1 and anti-SDHB antibodies, as lysosome and mitochondrial markers, respectively, we found that the two types of subcellular components are present in TnTs of SCC cells (Figure 5A). DAPI-stained particles were also found inside TnTs. Thus, we sought to determine whether mitochondria and/or lysosomes are exchanged between connected cells via TnTs. To this end, cells were labeled with the Celltracker green CMFDA plus either lysotracker or mitotracker. Figure 5B and Supplementary Video 4 illustrate the time-lapse sequence of images demonstrating the migration of lysotracker-containing acidic vesicles along the TnTs with an average migration rate of about 8 ± 1 nm/s. This traffic was detected in all TnTs recorded in SCC2 cells which contain mixed cell populations of epithelial- or mesenchymal-like phenotypes thus suggesting that it is not a specific feature of any of these phenotypes (data not shown). We also detected mitochondria cell-to-cell transfer along the TnTs with an average migration rate of about 6 ± 1.4 nm/s (Figure 5B and Supplementary Video 5).

**Figure 6:**
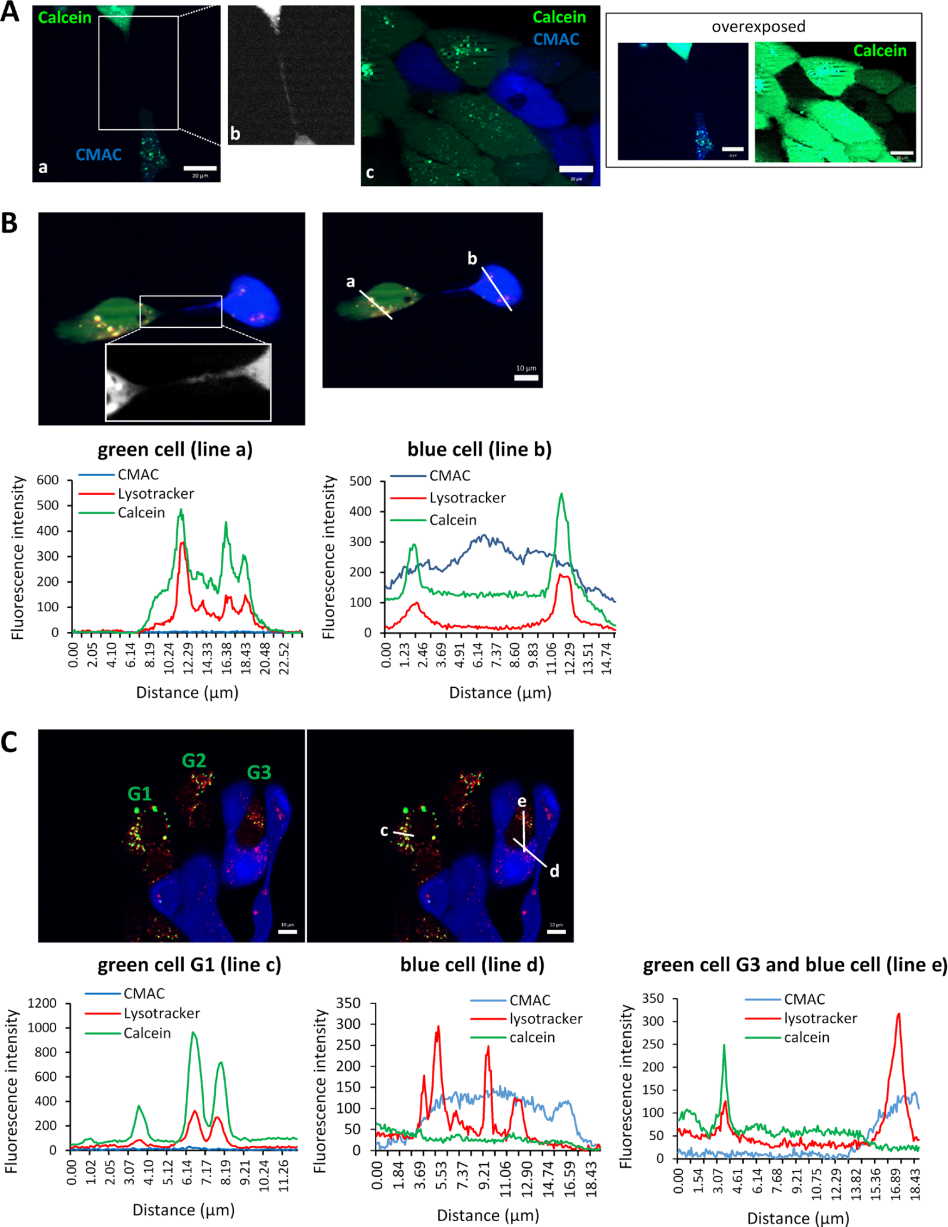
Traffic of calcein-AM-loaded lysosomes inside TnTs (**A**) SCC38 cells labeled with CellTracker blue CMAC were mixed (1:1) with SCC38 cells loaded with 0.5 μM calcein AM (green) and incubated at 37°C for 48 h before analysis. Panel b shows a magnified image of the area indicated in panel a. This image was transformed to black and white pseudocolor and was over-exposed to better visualize the blue TnT. Panel c shows the absence of transfer of blue or green dyes between adjacent cells not connected by TnTs. Over-exposed pictures are shown in the right to highlight that granular calcein-AM staining is not found in blue cells adhered to adjacent green cell. Scale bars, 20 μm. (**B**, **C**) Representative images showing colocalization imaging studies of calcein-AM and lysotracker. SCC38 cells labeled with CellTracker blue CMAC were mixed (1:1) with SCC38 cells loaded with 0.5 μM calcein AM (green) and incubated at 37°C for 48 h. Co-cultures were subsequently labelled with lysotracker (red) before analysis. A magnified image of the area indicated in panel B is shown to better visualize the thin TnT. Graphics show the fluorescence intensity profiles of the ROIs (a-e) indicated in the pictures. Calcein-AM labelled cells in C are denoted as G1-G3. Note that graphic in the left show colocalization of red and green fluorescence in the G3 cell that do not emit blue fluorescence while this colocalization is not observed in the cell emitting blue fluorescence. Scale bars, 10 μm.

Transfer of the fluorescent dye calcein acetoxymethyl ester (AM) from cells labeled with this dye to cells labeled with CellTracker Blue CMAC was also explored. Calcein-AM is a fluorogenic, cell-permeant acetoxymethyl ester derivative that is hydrolyzed by intracellular esterases in the cytosol to remove the acetomethoxy group, such that the molecule gets trapped inside the cell and emit green fluorescence. In our experimental conditions, calcein-AM was detected as diffuse cytoplasmic green staining as well as in shape of intensively stained granular structures. Transfer of cytoplasmic calcein-AM was difficult to evaluate given that during the time required for the cells to adhere and form TnTs, subtle calcein-AM transfer is found between both, adjacent cells and cells connected via TnTs. Although the intensity of the green fluorescence seemed to be higher in cells connected by TnTs than in adjacent cells, a definitive prove of calcein-AM transfer through TnTs could not be unequivocally demonstrated. By contrast, calcein-AM contained within granular structures was found in blue CMAC-stained cells that were connected with calcein-AM-loaded cells via TnTs (Figure 6A and 6B) but not in blue cells that were adhered to adjacent green cells (Figure 6C). This alerted us about the possibility that those structures actually could correspond to organelles such as lysosomes where calcein could have been accumulated during the course of experiments. Actually, the calcein-AM that has not been hydrolyzed in the cytosol may translocate to mitochondria or lysosome [26]. Thus, we tested this possibility by performing co-culture experiments in which blue CMAC cells co-cultured with calcein-AM cells were labelled with lysotracker (red). As shown in Figure 6, we confirmed that calcein-AM accumulates inside lysosomes and that the calcein-AM-loaded lysosomes are transferred to other cells via TnTs. Blue cells connected through a TnT to a green cell contains green+red dots (lysosomes loaded with calcein) (Figure 6B) whereas in the absence of TnTs, blue cells contacting green cells have only red dots (lysosomes that do not contain calcein-AM) (Figure 6C). Thus, the most plausible conclusion is that calcein-AM can be transferred through TnTs when loaded into lysosomes. Nevertheless, these experiments do not allow concluding whether cytoplasmic calcein-AM is transferred from cell-to-cell through TnTs.

**Figure 7:**
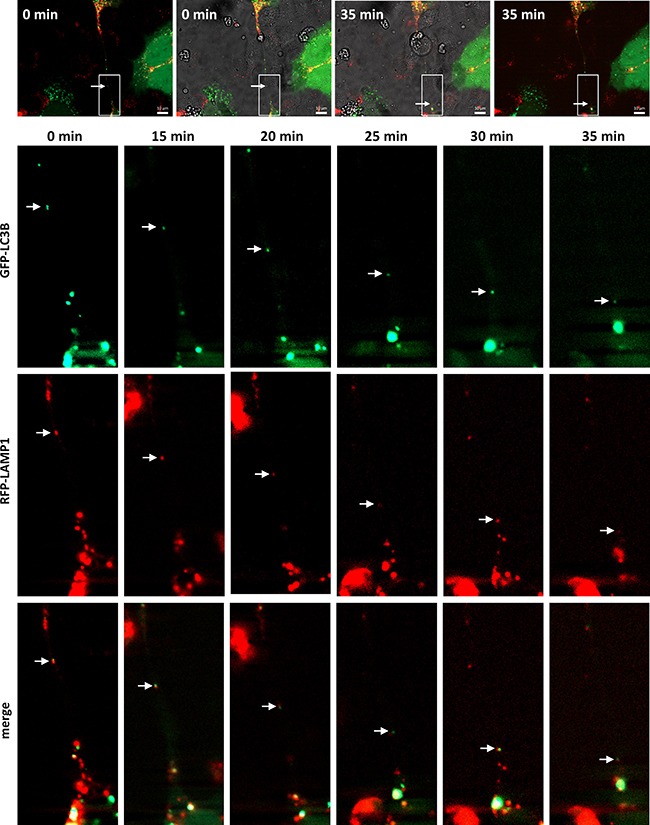
Traffic of autophagosomes, labeled with LAMP1 and LC3B, inside TnTs Representative images from time-lapse movies at the indicated time points. SCC38 cells were transiently co-transfected with LAMP1-mRFP-FLAG and pMXs-IP-EGFP-LC3B constructs. Merged transmitted light and fluorescence images at time 0 and 35 min are shown in the upper part of the Figure to illustrate the presence of the TnT connecting the cells and to point to the areas magnified in pictures below. White arrows denote red+green organelle. Scale bars, 10 μm.

**Figure 8:**
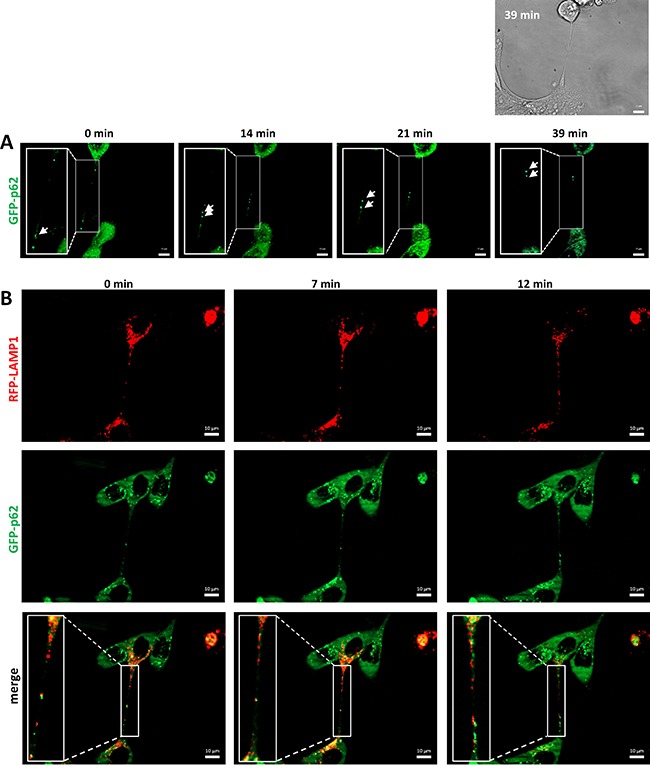
Traffic of autophagosomes, labeled with LAMP1 and p62, inside TnTs Representative images from time-lapse movies at the indicated time points. SCC38 cells were transiently transfected with pMXs-puro GFP-p62 (**A**) or co-transfected with LAMP1-mRFP-FLAG and pMXs-puro GFP-p62 (**B**) constructs. Bright-field image at time 0 and 39 min is shown in the upper part of panel A to illustrate the presence of the TnT connecting the two cells. Scale bars, 10 μm.

Lysosomes are versatile organelles that have emerged as key players of cancer progression to metastatic disease. For instance, they are responsible for recycling the cellular macromolecules that are delivered to them by autophagosomes [27]. Autophagosomes are generated as a strategic survival response that recycles energy and nutrients under special conditions such as hypoxia, stress and nutrient deprivation. Immunofluorescence analysis of an autophagosome marker, LC3A, and LAMP1 shows the presence of autophagosomes in close contact to the lysosomes located inside the TnTs (Figure 5A). To determine whether TnTs are used for cell-to-cell transfer of autophagosomes *in vivo*, SCC38 cells were co-transfected with two constructs, one expressing LC3B fused to eGFP and another expressing LAMP1 fused to mRFP. As shown in Figure 7, the lysosomal and autophagosomal makers co-localized inside the TnTs and moved directionally from one cell to another along time. We also analyzed the movement of another autophagosomal marker, the LC3-binding protein p62. Cells transfected with a construct expressing GFP-p62 revealed the presence of this marker inside the TnTs (Figure 8A). As expected, GFP-p62 co-localized with mRFP-LAMP1 in co-transfected cells. These combined organelles were found densely concentrated inside the TnTs and similarly to the LC3B+LAMP1 vesicles showed unidirectional movement between the two connected cells (Figure 8B).

### Motion of rhodamine-loaded polymeric nanoparticles along TnTs

Because TnTs may represent cancer-specific vulnerabilities that could be exploited therapeutically, we sought to determine whether drug nanocarriers were able to travel along TnTs. To this end, we constructed inert polymeric nanoparticles of 142.5 nm of mean diameter which were loaded with Rhodamine 6G for their visualization by fluorescence microscopy. These nanoparticles were efficiently and spontaneously internalized into cells and lacked any cytotoxic effect (data not shown). Time-lapse experiments revealed a scattered pattern of subcellular distribution that extended to TnTs, (Figure 5Ak) where nanoparticles moved from one cell to another (Figure 5C and Supplementary Video 6). We ruled out that nanoparticles are contained within lysosomes. As shown in Supplementary Figure 5, nanoparticles do not colocalize with autophagolysosomal GFP-LC3B in SCC38 life cells neither with lysosomal LAMP1 or mitochondrial SDHB in immunofluorescence analysis. Further studies are underway to use drug-loaded nanoparticles traveling along TnTs as drug delivering systems, an approach that holds great promise for cancer treatment.

### Role of actin microfilaments and microtubules on TnT formation

**Figure 9:**
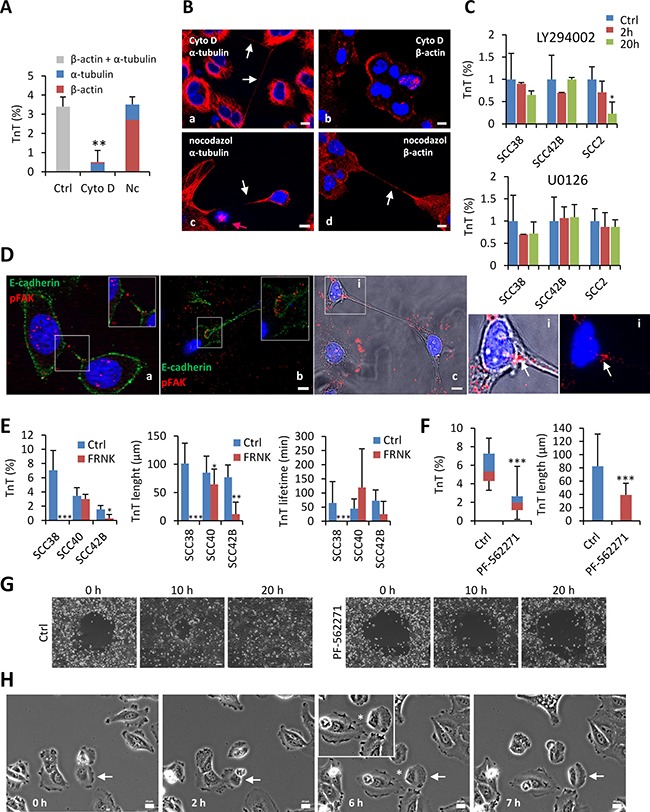
Involvement of actin microfilaments, PI3K and FAK in TnT formation (**A**) Percentage of SCC38 cells containing TnTs upon treatment with solvent (Ctrl, 0.1% DMSO), cytochalasin D (Cyto D, 1 μM) or nocodazole (Nc, 1 μM) for 24 h. The percentages of TnTs that contains β-actin or α-tubulin after treatments were quantified by immunofluorescence staining with the corresponding antibodies. Values are mean ± standard deviation from two independent experiments (3246 cells counted). (**B**) Representative images of the β-actin and α-tubulin immunostainings in cells treated with cytochalasin D or nocodazol. White arrows indicate TnTs. Note that treatment with nocodazole efficiently induced cell cycle arrest (pink arrow in c) but did not inhibit TnT formation. Scale bars, 10 μm. (**C**) Quantification of the percentage of TnTs relative to the total number of cells. Cells were treated with solvent (0.1% DMSO), U0126 (5 μM), or LY294002 (25 μM) for the indicated times. Values are mean ± standard deviation from two independent experiments (1500-2000 cells counted in each). (**D**) Representative images of staining for E-cadherin and pY397 FAK (pFAK) (a,b) and pY397 FAK (c) in SCC42B cells. Insets show that pFAK immunostaining appears as small dots in some TnTs and as focal-adhesion-type linear structure in others. Image c is a color composite image of bright field and fluorescence. Panels i show magnified images (bright field and fluorescence on the left picture and only fluorescence on the right picture) of the indicated area. White arrows in (i) indicate pFAK staining of focal-adhesion-type linear structures at the edge of the TnT. Scale bar, 10 μm. (**E**) Quantification of the percentage of TnTs relative to the total number of cells, and the TnTs maximal length and lifetime in control (Ctrl) and FRNK-SCC cells (550 cells in each condition counted by using bright field microscopy) (**F**) Quantification of the percentage and the length of TnTs relative to the total number of cells in cells treated or not with PF-562271 (1 μM) for 24 h. Values are mean ± standard deviation from two independent experiments (1500-2000 cells counted in each). Measurements were performed using bright field microscopy. * indicates *P* < 0.05, ** indicates *P* < 0.005, *** indicates *P* < 0.0005. (**G**) Representative images of wound healing assay performed in SCC42B cells in the presence of 0.1% DMSO (Ctrl) or 1 μM PF-562271 at the indicated time points. Scale bars, 100 μm. (**H**) Representative images of FRNK-SCC40 cells from time-lapse movies at the indicated time points. White arrows point to a cell involved in the assembly of a TnT.

To get insights into the molecular mechanisms involved in the TnT assembly, we first analyzed the role of actin and microtubule polymerization. Inhibition of actin polymerization by cytochalasin D significantly repressed (86% inhibition) the formation of TnTs in SCC38 cells (Figure 9A). Immunostainings with α-tubulin or β-actin showed that, in the presence of cytochalasin D, 86% and 14% of the TnTs that were assembled contained α-tubulin and β-actin, respectively (Figure 9A and 9B). In contrast, microtubule-disrupting agents, such as colchicine (data not shown) or nocodazol (Figure 9A), did not block TnT formation even when used at high dosage (1 μM). Most TnTs formed under these conditions contained β-actin. TnTs assembled in cells treated with cytochalasin D or with nocodazol did not allow transfer of lysotracker-labeled vesicles although some movements of vesicles were detected (data not shown). Thus, TnT formation requires efficient actin polymerization whereas both, actin and microtubules, are required for the cell-to-cell transfer of vesicles.

PI3K and MAPK have active roles in actin filament remodeling and have been suggested to be involved in the regulation of TnT formation [28]. However, we found that they are dispensable for TnT formation in SCC cells. As shown in Figure 9C, the PI3K- and MAPK-specific inhibitors, LY-294002 and U0126, did not reduce TnT densities in SCC38 and SCC42B cells. We only detected a 75% reduction of TnTs in SCC2 cells treated with LY-294002 for 20 hours. This suggests that PI3K regulates TnT formation in a cell-type specific manner and that other actin cytoskeletal remodeling mechanisms must be involved in TnT formation.

### Regulation of TnT by FAK

We hypothesized that FAK, which links the ECM and the intracellular actin system, could be involved in TnT formation/stabilization. To this end, we used previously generated FRNK-SCC38 and FRNK-SCC40 cells in which the FAK-mediated signal transduction was disrupted by expression of FRNK, a truncated isoform of FAK [29, 30] that acts as a dominant negative and blocks FAK-mediated cell migration, invasion and MMP-2 activation [31]. For the present studies, we also generated FRNK-SCC42B cells, which, as FRNK-SCC38 and FRNK-SCC40 cells, showed reduced cell migration (Supplementary Methods and Supplementary Figure 6). Imunofluorescence of pY397 FAK revealed the presence of active pFAK at the end of the TnTs in SCC38 and SCC42B cells (Figure 9D).

To test our hypothesis, we quantified the percentage of cells containing TnTs in control (Ctrl)- and FRNK-cells. TnTs almost completely disappeared in FRNK-SCC38 cells and were significantly reduced in FRNK-SCC42B, in comparison with their respective Ctrl-cells (Figure 9E). In contrast, the TnT density was not altered in SCC40 cells upon FRNK expression, although they had shorter length than those of Ctrl-SCC40 cells (see Figure 9E, 9H). TnT shortening was also observed in FRNK-SCC42B and FRNK-SCC40 cells. TnT lifetime was significantly reduced in FRNK-SCC42B cells, but did not change in FRNK-SCC40 cells in comparison with their respective controls (Figure 9E). Thus, shortening of the TnTs, rather than decreased lifetime or density, is the common phenotype found in the three cell lines upon FRNK expression.

We next used a pharmacological approach in which we tested the effect of PF-562271, a FAK tyrosine kinase inhibitors that is currently undergoing clinical testing, on TnT formation in SCC cells [32]. We first studied the inhibitory efficiency of PF-562271 by analysis of SCC cell motility. As shown in Figure 9G, PF-562271 decreased the migratory potential of SCC42B cells measured by wound healing assay. Analysis of TnT formation showed that, as found in FRNK-cells, TnT length and percentage decreased in SCC cells treated with 1 μM PF-562271 (Figure 9F). These data showed that functional FAK regulates the ability of cells to establish long distance cellular contacts through TnTs.

### Restoring defective intercellular communication through TnTs in FRNK-SCC cells

**Figure 10:**
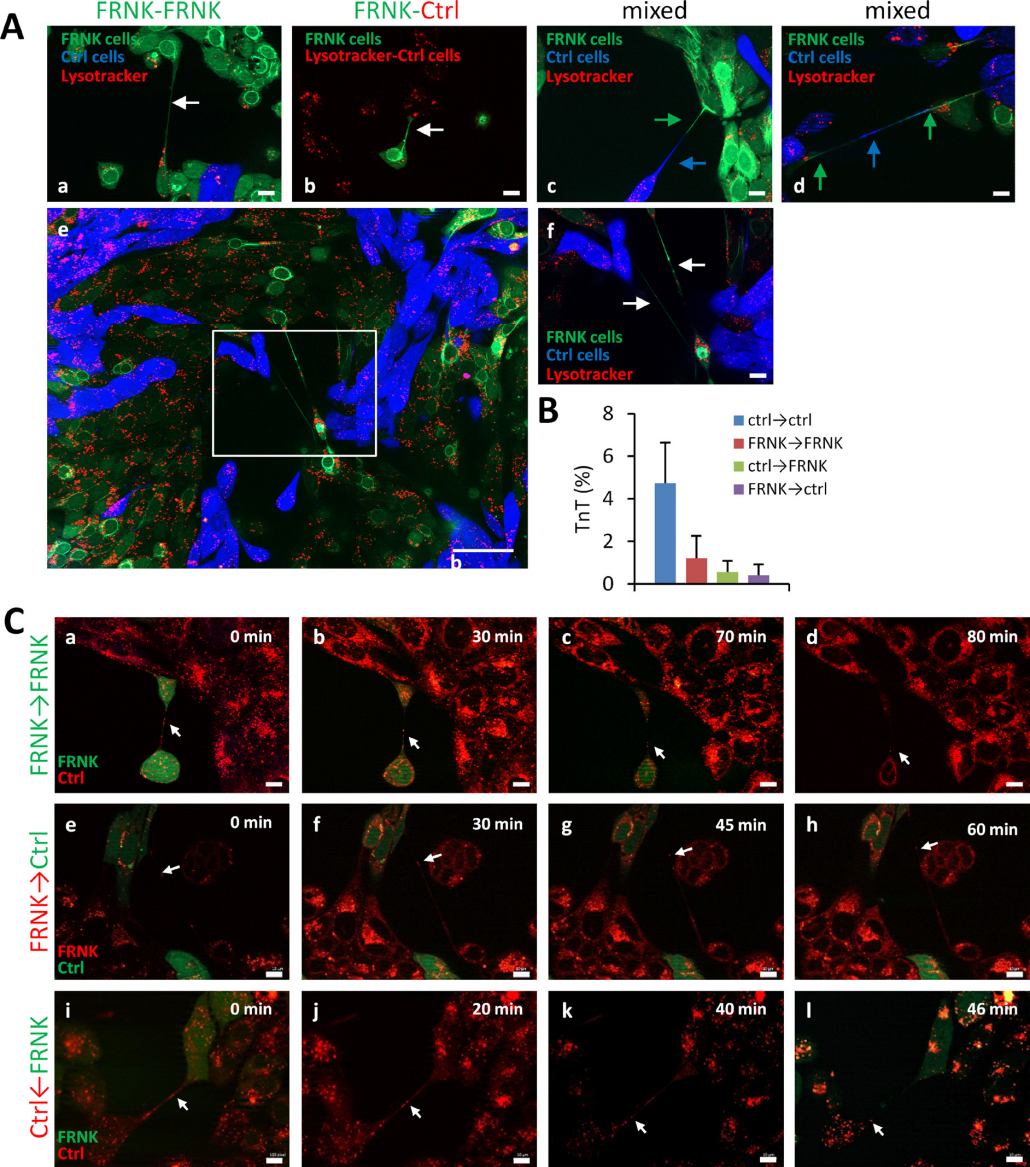
Traffic of endosomal/lysosomal vesicles through TnTs that connect FAK-proficient and FAK-deficient cells (**A**) Representative images of FRNK- and Ctrl-cells co-cultures. FRNK-SCC38 and Ctrl-SCC38 cells were labeled with CellTracker green CMFDA and CellTracker blue CMAC dye, respectively (a, c, d). In panel b, Ctrl-cells were not labeled. Co-cultured cells were subsequently labeled with lysotracker (red). Image in (e) was taken by using a 3 × 3 tile. Stitching was performed with the Zen software. (f) Higher magnification of the area outlined in the image (e). White arrows denote FRNK-FRNK TnTs (a, f) and FRNK-Ctrl TnTs (b). Mixed, green and blue, TnTs are indicated by green and blue arrows (c, d). (**B**) Quantification of TnTs that emerge from Ctrl-cells and contact with Ctrl (Ctrl-Ctrl) or FRNK cells (Ctrl-FRNK) and TnTs that emerge from FRNK and contact with Ctrl (FRNK-Ctrl) or FRNK cells (FRNK-FRNK). Data are presented as mean ± standard deviation from 2 individual experiments and 2000 cells analyzed in each. Measurements were performed using fluorescence microscopy. (**C**) Representative images from time-lapse movies at the indicated time points. FRNK-SCC38 cells labeled with green CMFDA were co-cultured (1:1) with non-fluorescently labeled FRNK- (a-d) or Ctrl-SCC38 cells (i-l). (e-h) Ctrl-SCC38 cells labeled with green CMFDA were co-cultured (1:1) with non-fluorescently labeled FRNK-cells. Co-cultured cells were subsequently labeled with lysotracker (red). White arrows denote lysotracker-labeled vesicles traveling through TnTs that connect FRNK-FRNK cells (a-d) or FRNK-Ctrl cells (e-l). Note that the direction of movement along FRNK-Ctrl TnTs is from the FRNK to the Ctrl-cell irrespective of whether the TnT emerge from a FRNK (e-h) or from a Ctrl-cell (i-l). Scale bars, 10 μm.

We next sought to determine whether FAK-proficient cells could rescue the aberrant phenotype of FAK-deficient cells. To this end, FRNK-SCC38 cells, labeled with CellTracker green CMFDA dye, were co-cultured with Ctrl-SCC38 cells labeled with CellTracker blue CMAC dye. The data revealed that, under these conditions, FRNK-SCC38 cells established close contacts with Ctrl-SCC38 cells, increased spread cell area, and recovered the elongated cell phenotype and the ability to emit TnTs which established contacts with other FRNK-cells and, less frequently, with Ctrl-cells (Figure 10A and 10B). We also detected TnTs emitted from Ctrl-cells and connecting with FRNK-cells. Nevertheless, the most frequent TnT connections were found between two Ctrl-cells. Although the great majority of TnTs harbored green or blue labeling, a few mixed blue+green TnTs were also detected (Figure 10Ac-d).

Staining of acidic vesicles with lysotracker showed the presence of endosomal/lysosomal organelles inside the TnTs emitted from FRNK-SCC38 cells in the co-culture experiments (Figure 10C). Co-staining of LC3A and LAMP1 revealed full co-localization of the two markers in FRNK-SCC38 cells (data not shown). Further, time-lapse recording revealed that lysosomes/ autophagosomes migrated along the TnTs from one FRNK-cell to the other, although with a migration rate slower (4.7 ± 1.4 nm/s) than that achieved in TnTs connecting Ctrl-cells. Interestingly, movement of these vesicles was also detected in TnTs emitted from FRNK- to Ctrl-cells and in TnTs emitted from Ctrl- to FRNK-cells. In these TnTs, the migration of lysosomes/autophagosome vesicles appeared to be unidirectional from FRNK- to Ctrl-cells irrespective of whether the TnT was emitted from a Ctrl-cell or from a FRNK-cell. Nevertheless, the migration rate through FRNK-TnTs was slower (3.65 ± 0.75 nm/s) than that through Ctrl-TnTs (9.38 ± 3 nm/s).

**Figure 11:**
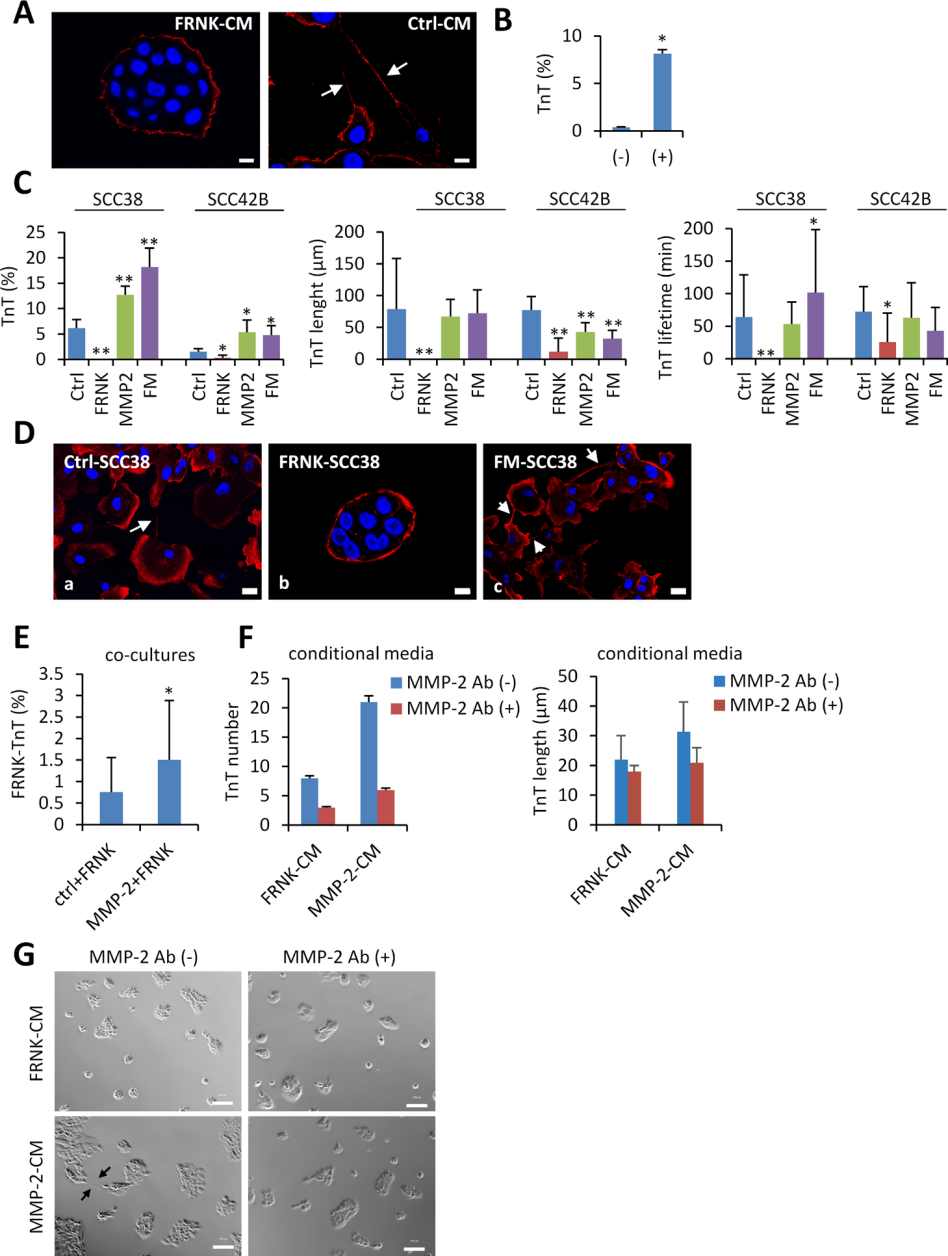
Rescue of TnT formation in FAK-deficient cells (**A**) Representative images of staining for β-actin in FRNK-SCC38 cells incubated for 24 h with conditioned medium obtained from FRNK-SCC38 cells (FRNK-CM) or Ctrl-SCC38 cells (Ctrl-CM). The conditional media were collected after 48h of cell culture at 70% confluence. White arrows denote TnTs. (**B**) Quantification of TnTs in FRNK-SCC38 cells co-cultured with FRNK-CM (−) or Ctrl-CM (+). Data are presented as mean ± standard deviation from 3 individual experiments and 200-400 cells analyzed in each. Measurements were performed using fluorescence microscopy. (**C**) Quantification of the percentage of cells with TnTs, and the TnT-maximal length and lifetime in the indicated Ctrl, FRNK-, MMP-2- and FRNK-MMP2 (FM)-cells. Measurements were performed using bright field microscopy. Data are presented as mean ± standard deviation from 3 individual experiments and 2500-3000 cells analyzed in each. (**D**) Representative images of staining of β-actin in the indicated cell lines. Note that FRNK-SCC38 cells lack TnTs but these re-appear in FRNK cells upon MMP-2 over-expression (FM-SCC38). (**E**) Percentage of TnTs in FRNK-SCC38 cells when co-cultured with Ctrl-SCC38 cells (Ctrl+FRNK) or with MMP-2-SCC38 cells (MMP-2+FRNK). To distinguish different cell types, Ctrl- or MMP2-SCC38 cells were labelled with green CMFDA and FRNK-SCC38 cells were labelled with blue CMAC. Measurements were performed using fluorescence microscopy. Data are presented as mean ± standard deviation from 2 individual experiments and 2500-3000 cells analyzed in each. (**F**) TnT numbers in FRNK-SCC38 cells incubated for 24 h with conditional medium obtained from FRNK-SCC38 cells (FRNK-CM) or MMP-2-SCC38 cells (MMP-2-CM) in the presence (+) or absence (−) of blocking MMP-2 antibody. The conditional media were collected after 48h of cell culture at 70% confluence. TnT counting was performed using bright field microscopy. Data are presented as mean ± standard deviation from 2 individual experiments and 20000 cells analyzed in each. (**G**) Phase contrast representative micrographs of FRNK-SCC38 cells incubated with the indicated conditional media in the presence or absence of MMP-2 antibody. Scale bars, 10 μm. * indicates *P* < 0.05, ** indicate *P* < 0.005.

To determine whether cell-cell contacts were required for the rescue effect observed in the co-culture experiments, FRNK-SCC38 cells were incubated with Ctrl-SCC38-conditional media. As shown in Figure 11A and 11B, a subtle but significant increase of TnTs between FRNK-cells were observed under these conditions thus indicating that a soluble factor secreted by Ctrl-cells and likely also cell-to-cell contacts are involved in the activation of TnT formation in FRNK-cells by Ctrl-cells.

To delineate signaling events connected with those phenotypes, we analyzed whether the MMP-2 metalloprotease is involved in the regulation of TnT formation by FAK. TnT densities, maximum length and lifetime in FRNK-SCC38 cells were compared with those of FRNK-SCC38 cells stably transfected with a MMP-2-expressing construct (FM-SCCs). Pooled clones of FAK-proficient cells stably transfected with MMP-2 (MMP-2-SCC) were also evaluated. As shown in Figure 11C and 11D, MMP-2 over-expression in SCC38 cells did not modify their ability to emit TnTs whose length and lifetime were similar to those of Ctrl-cells. However, MMP-2 over-expression in FRNK-cells notably increased TnT formation reaching levels similar to those detected in Ctrl-cells. These TnTs were similar in length to TnTs of Ctrl-cells and seemed to be more stable in culture with a longer lifetime (101 ± 96 min) than TnTs of Ctrl-cells (64 ± 64 min). Here, we also generated MMP-2-SCC42B and FM-SCC42B cells (Supplementary Figure 6). In SCC42B cells, MMP-2 expression induced a 3.5-fold increase of TnT density and a 1.8-fold decrease in TnT length, but did not significantly alter their lifetime. Similarly to SCC38 cells, MMP-2 over-expression in FRNK-SCC42B cells reversed the inhibitory effect of FRNK on TnT formation and allowed formation of TnTs with features similar to those of Ctrl-SCC42B and MMP-2-SCC42B cells with regard to their length and lifetime.

MMP-2-SCC38 cells were then used for co-culture experiments with FRNK-SCC38 cells. As it was observed in Ctrl-SCC38+FRNK-SCC38 co-culture experiments, FRNK-SCC38 cells recovered the elongated cell phenotype and the ability to emit TnTs in the presence of MMP-2-SCC38 cells being this effect greater than that observed in the presence of Ctrl-SCC38 cells (Figure 11E and Supplementary Figure 7). The FRNK-induced cellular phenotype was also partially reversed upon incubation of FRNK-SCC38 cells with MMP-2-SCC38-conditional media, but not when the conditional media was pre-incubated with neutralizing antibodies against MMP-2 (Figure 11F and 11G).

## DISCUSSION

This work reveals the presence of TnTs in head and neck SCC, describes a new mechanism of direct exchange of cargos between distant SCC cells via TnTs and discloses a not previously identified function for FAK and MMP-2 in the formation of these novel cellular structures.

### Identification of TnTs in SCC cells as new routes for organelle cell to cell transfer

Since their initial description in PC12 cells, TnTs have received several names in the scientific literature (cytonemas, intercellular nanotubes, filopodial bridges, and, more recently, tumour microtubes to define ultra-long, long-lived, and thick membrane extensions found in astrocytoma cells [13]) and have been shown to harbor different morphological and molecular properties in distinct cell types [33]. For those reasons, we considered of great importance characterizing in detail the cellular protrusions identified in SCC cells and to compare them, in our experimental conditions, with those described in PC12 cells [14]. Recent review reports have concluded that there may be two types of TnTs (4): (a) PC12-like TnTs that are thin (< 0.7 μm) and contain actin, but not microtubules, and (b) thick TnTs (> 0.7 μm) that contain both actin and microtubules. Interestingly, these thick TnTs have been recently identified in stressed PC12-cells [11]. We show here that SCC-TnTs, as PC12-TnTs, set direct connections between distant cells, hovered freely in culture medium and were induced by serum deprivation in the culture media. However, in contrast to the initially described PC12-TnTs, we found that SCC-TnTs were thicker, longer and more persistent. Indeed, the SCC-TnTs are among the longest TnTs reported thus far [13, 33]. Further, SCC-TnTs contained actin and microtubules. Thus, we concluded that SCC-TnTs were similar to those recently reported in stressed PC12 cells and other cancer cell types, as human lung carcinoma cells [34] and astrocytomas [13]. The TnTs identified here are also similar to one of the structures (called TT5) described by Antanaviciute et al. in laryngeal carcinoma cells [18]. That report describes other types of projections that establish contacts between cells which have not been identified in the four SCC cell lines that we analyzed.

Although many studies have ascribed physiological or pathological functions to TnTs based on analysis on cultured cells, there are very few evidences documenting TnTs *in vivo* due to the technical difficulties associated to the lack of specific molecular markers [4]. Because two-dimensional tumor cell line cultures fail to recapitulate the three-dimensional context of cells in solid tumors, we looked for the presence of TnTs in tumor spheroids, which remain the best characterized and most widely used three-dimensional model [25]. We report here that SCC-TnTs can be formed inside tumor spheroids. Further, although we cannot conclusively demonstrate the presence of TnTs in tumour tissues, long actin and tubulin filamentous structures connecting tumor cells could also be visualized in human tumor tissues and in orthotropic tumor xenograft models of head and neck SCC. Future studies will be required to definitively demonstrate the presence of TnTs *in vivo*, once specific markers become available. Hitherto, the data presented in this report strongly support the idea that TnTs are formed in squamous cell carcinomas and are not a consequence of the artificial conditions to which cultured cells are subjected.

Another interesting data revealed in this report is that the SCC-TnTs do not seem to be a specific feature of mesenchymal or epithelial cells, as they were detected in CK^+^E^+^ cells and VIM^+^N^+^ cells. Furthermore, promiscuous monotypic and heterotypic contacts via TnTs were found in culture. This suggests that, within the intricate areas of metastatic tumors, cells undergoing the epithelial to mesenchymal transformation, required for the acquisition of an invasive behavior, can still maintain direct communication with the less invasive distant epithelial cells. The type/s of specific signal/s that is transmitted along these TnTs and their functional consequence/s are still unknown. TnTs have been proved to allow for direct transfer of cellular components such as mitochondria, endosomal/lysosomal vesicles, proteins, microRNAs and Ca^2+^ ions [5–10, 12, 24]. Fluorescence microscopy of labeled organelles has allowed us to identify mitochondria and endosomal/lysosomal vesicles fused to autophagosomes inside the SCC-TnTs. We have also visualized the net transfer of the autophagosome/lysosomal vesicles and mitochondria thus indicating that TnTs are used a routes for organelle transfer between cancer cells. Different studies have shown than TnTs develop from insulted to non-insulted cells [11, 22, 35]. Given the plethora of proteins located within or shuttled to the endosomal/lysosomal system, autophagosomes and mitochondria, it would not be surprising that many of the components of these organelles could participate in crucial cellular processes, such as cell growth, survival and death decisions and that TnTs represent a route to get rid of or capture these signals.

We also show that TnT formation is not a specific feature of cancer cells since can also be found in CAFs derived from human SCCs. Morphologically, these TnTs are similar to those of SCCs cells except for their length, TnTs of CAFs connect cells across shorter distances than those assembled in SCC cells. Furthermore, co-culture of SCC cells and CAFs showed that although some TnTs from CAFs can connect with SCC cells and vice versa, cell-to-cell communications through TnTs seem to choose preferentially cells of the same type. In-depth molecular and functional characterization of CAF- and SCC-TnTs is mandatory if these structures are considered as therapeutic cancer targets.

### Regulation of TnT assembly in SCC cells by FAK and MMP-2

The molecular basis for TnT formation is far from being fully understood [28, 36]. The present report provides a novel piece of information towards a comprehensive model of the molecular mechanisms and factors affecting the formation of TnTs. We show that cytochalasin D-induced impairment of actin polymerization inhibited TnT formation although a few α-tubulin-containing TnTs could still be assembled. By contrast, TnT formation was not affected by nocodazol, a microtubule-disrupting agent. The TnTs assembled in the presence of nocodazol mainly contained β-actin, thus indicating that actin filaments, but not microtubules, are essential components of the TnT assembly machinery. We also show that PI3K but not MAPK activities, that have active roles in actin filament remodeling, may be involved in TnT assembly but in a cell-type specific manner. However, by using FRNK, a dominant negative inhibitor of FAK, we disclose that functional FAK is required for proper TnT formation in all cell lines that were tested. FAK is at the center of the dynamic interplay between integrins-ECM and cell-cell adhesion [37], both of them closely related to changes in actin remodeling and actomyosin contractility. Thus, impairment of actin- or ECM-remodeling or increase of cell-cell adhesive strength following FAK inhibition could be the molecular basis for inhibition of TnT formation. Alternatively, because cell migration is severely impaired in FRNK-SCC cells [31], it is possible that the effect of FAK inhibition on TnT formation is the result of fewer opportunities of the cells to establish contacts with other cells and move apart. Although this possibility cannot be completely ruled out, the absence of correlation between decreased TnT formation and reduced migratory capacity of the cells suggests a direct link of FAK and TnT assembly. For example, cell migration, but not TnT density, decreased in FRNK-SCC40 cells versus Ctrl-cells. In addition, cell migration slightly decreased in MMP-2-SCC42B versus Ctrl-SCC42B cells but TnT density increased. In contrast, shortening of the TnT length is the common phenotype found in the three cell lines upon FAK inhibition. These observations lead support to the notion that FAK regulates TnT assembly via its involvement on actin- or ECM-remodeling, or cell-cell adhesion rather than being an indirect consequence of reduced cell migration. This was not surprising based on the strong consensus evidences indicating that FAK is involved in the malignant conversion to invasive carcinoma likely as a consequence of, among other cues, the deregulation of actin cytoskeleton dynamics [38]. The novelty of our work is that the FRNK-induced inhibition of TnT formation is partially reversed by MMP-2. Our previous work demonstrated that the inhibition of FAK activity severely reduces cell migration and invasion via inhibition of MMP-2 [31]. Here, we found a reversion of the FRNK effect on TnT formation by MMP-2 over-expression in FRNK-cells. Co-culturing of FRNK-cells with FAK-proficient cells led to a partial recovery of the ability of FRNK-cells to emit TnTs and this effect was further increased when FRNK-cells were co-cultured with FAK-proficient cells over-expressing MMP-2. Finally, conditioned-culture media from cells over-expressing MMP-2 induced TnT formation from FRNK-cells, an effect that was blocked by MMP-2 neutralizing antibodies. Similar results were obtained when conditioned-culture media was from FAK-proficient cells although this effect was of less magnitude than that observed with conditioned media from FAK-proficient cells stably expressing MMP-2. Collectively, we uncovered a novel function for the FAK/MMP-2 signaling axis in cell-cell communication via TnT formation. Interestingly, TnTs emanating from FRNK-cells in the co-culture experiments established connections with other FRNK-cells, or with FAK-proficient cells over-expressing or not MMP-2, and contained endosomal/lysosomes/autophagosome vesicles that migrated unidirectionally from FRNK- to FRNK-, to Ctrl-, or to MMP-2-cells. Migration of the acidic vesicles was also found along TnTs that emanated from Ctrl- or MMP-2-cells and ended in FRNK-cells. In these TnTs, the migration of vesicles was also found to be unidirectional, from FRNK-cells to Ctrl- or MMP-2-cells. Further studies are required to determine whether the trafficking of organelles is intended to get rid of or capture specific cellular signals. Recently, Sandilands et al. showed the autophagic targeting of active Src in cells with impaired integrin/FAK signalling pathway [39]. We show here that, under these circumstances, cells tend to get rid of autophagosomes. Collectively, the data suggest that both, the autophagosome targeting of FAK-associated proteins and the autophagosome transfer from FAK-deficient to FAK-proficient cells may be used as a route to survive microenvironmental stress elicited by impaired flux through the FAK pathway and to increase growth and aggressiveness.

Although the role of TnTs in cancer development and/or tumor dissemination is not known, the recent report of Osswald et al. [13] showing that astrocytoma cells use long membrane protrusions, morphologically similar to those reported here, as routes for brain invasion and proliferation suggest that disruption of TnTs may benefit the clinical course of cancer patients. Importantly, treatment of SCC cells with PF-562271, a clinically relevant FAK inhibitor, significantly impairs TnT assembly. There are several recently completed or ongoing early-phase clinical trials using FAK inhibitors to treat patients with solid tumors (http://clinicaltrials.gov/; NCT00666926, NCT00996671, NCT00787033, NCT01138033). Although it is still too early to draw conclusions from these clinical trials, several patients had disease response while on treatment with PF-562271 [40]. Our study supports further investigation to determine the role of TnTs in cancer metastasis, cancer cell survival, and of FAK/MMP-2 axis as a therapeutic target for disruption of communication of cancer cells via TnTs. Additionally, once the biochemical intersection of FAK and autophagy flux have been unraveled, inhibition of both autophagy and FAK-signaling could represent a novel strategy to improved cancer therapy results.

Finally, data presented in this communication should raise the idea that the formation of TnTs, besides representing a new route that could greatly enhance cellular communication in human neoplasms, could also be exploited therapeutically as drug-delivery channels for cancer therapy. We show that polymeric nanoparticles can be efficiently loaded within TnTs and move along these channels able to connect epithelial cancer cells and less differentiated more invasive mesenchymal cancer cells. These data highlight new vulnerabilities that can be exploited to efficiently eradicate cancer cells by the use of drug-loaded nanoparticles trafficking thorough TnTs as drug delivering systems, an approach that holds great promise for cancer treatment.

## MATERIALS AND METHODS

### Cell culture and transfections

The established human squamous cell carcinoma (SCC)-derived cell lines were kindly provided by Dr R Grenman (University Central Hospital, Turku, Finland). Cells were grown as previously described [31]. All SCC cell lines were periodically tested for human pathogens and mycoplasma infection. Transient transfections and generation of FRNK- and FRNK-MMP-2-SCC42B cells were performed as described in Supplementary methods. All methods were carried out in accordance with the approved guidelines of our institution.

For lentiviral infections, 293T cells were transiently co-transfected with lentiviral packaging mix and LAMP1-mRFP-FLAG (Addgene) using Lipofectamine 3000 reagent. For retroviral infections, Phoenix-Ampho packaging cells were transfected with pMXs-IP-EGFP-LC3 or pMXs-puro GFP-p62 (Addgene) plasmids using Lipofectamine 3000 reagent. The virus-containing media were collected 48 h post-transfection and immediately used to infect SCC38 cells in the presence of 4 μg/ml of polybrene for 24 hours. The infection procedure was repeated 24 h later.

### TnT identification and quantification

For TnT counting, cells were grown until they reached about 70% confluence which was found as the optimal confluence to allow TnT formation. Only one type of long membrane projection that connects remote cells was identified in SCC cells and these fulfilled all the criteria to be defined as TnTs as their presence in middle or upper stacks of the picture in Z dimension and the absence of midbodies which are present in the cytokinesis bridges that connect cells at the final step of cell division [41]. This aspect simplified the identification and counting of TnTs ruling out any confusion with other cell bridges. TnT quantifications were achieved by using bright field or fluorescence microscopy in life-cells or fixed-cells as indicated in each Figure legend. For estimations of TnT percentages and length, cells were pictured using 8 × 8 tiles. Although time-lapse video microscopy revealed that SCC cells formed TnTs by retaining a progressively thinner and longer thread of membrane upon cell dislodgement, only long (> 10 μm) membrane bridges were considered for TnT quantifications. Means were calculated and compared using two-sided, two-tailed *t*-tests. *P* values ≤ 0.05 were considered statistically significant.

### Polymeric nanoparticles

Polymeric nanoparticles based in a monomer mixture of methyl methacrylate/2-hydroxymethyl methacrylate/glycidyl methacrylate in a weight proportion of (70:20:10) were synthesized following the protocol previously described [42]. Sodium dodecyl sulfate was used as colloidal surfactant stabilizer. Rhodamine 6G (Exciton), in a concentration of 5 × 10^−4^ M, was incorporated into a colloidal suspension of nanoparticles (5% weight proportion) by free diffusion. The size distribution by volume of the sensitized nanoparticles, determined by dynamic light scattering, was centered at about 142 nm and had a relative standard deviation of 0.11-0.12.

### Fluorescent cell labeling and co-cultures

Cells were labeled with the fluorescent dyes CellTracker^™^ Blue CMAC 7-amino-4-chloromethylcoumarin or CellTracker™ Green CMFDA 5-chloromethylfluorescein diacetate (Thermo Fisher) following manufacturer's instructions. Organelles were labeled using the LysoTracker^®^ Red DND-99 or MitoTracker^®^ Red CMXRos (Thermo Fisher) following manufacturer's instructions. For co-culture experiments, equal amounts of cells, fluorescently labelled or not as indicated in each Figure legend, were seeded and pictured and/or time-lapse recorded using Zeiss AxioObserver Z1 with AxioCam MRM and ApoTome 2 (Carl Zeiss, Germany) 12–14 hours after seeding. For the analysis of trafficking of organelles, automated video recording could not be used due to the dynamism of the TnTs which move over time not only in the x-y but also in the z plane. Thus, images were focused before each picture taken every 5 minutes, a strategy that also helped to avoid photobleaching. Moreover, only TnTs that did not significantly change their length during the recording time were used for quantification of velocity of organelle movements. Time-lapse sequences of trafficking organelles were arranged to create a movie using Image J- Fiji. For analysis of calcein cell-to-cell transfer, SCC38 cells were seeded and allowed to adhere for 10 hours before labelling with calcein AM (0.5 μM for 30 min) followed by thorough washes. Then, an equal amount of SCC cells, labeled with blue CMAC, was added to the cell culture. Co-cultured cells were examined 48 hours later.

### Tumor-spheres generation

For tumor-spheres generation, cells were cultured in a spheroid formation media (growth culture medium supplemented with 0.2% methylcellulose) in non-adhesive convex environment for 12 h at 37°C and 5% CO_2_. Tumor-spheres were mixed with collagen matrix (2.5 mg/ml) and incubated for 30 min at 37°C prior to microscopic analysis.

### Immunocytochemistry

Immunostainings of cells or tissues were performed as previously described [31] with anti-β-actin and anti-α-tubulin (Sigma-Aldrich), anti-cytokeratin and anti-N-cadherin (DaKo, Agilent Technologies), anti-E-cadherin, anti-human FAK (pY397) and anti-clathrin heavy chain (BD Transduction Laboratories^™^), and anti-vimentin (Abcam).

### Time-lapse and confocal reflection microscopy

Time-lapse microscopy imaging was performed on a Zeiss AxioObserver Z1 microscope (Carl Zeiss, Germany) with a Plan-Apochromat 40X/1.3 (NA = 1.3, working distance = 0.21 mm) or Plan-Apochromat 63X/1.4 (NA = 1.4, working distance = 0.19mm) oil lens objective, a camera (AxioCam MRm; Carl Zeiss), and Apotome (ApoTome 2; Carl Zeiss). z-stack images were taken with AxioVision module Z-stack (Zeiss). Three-dimensional reconstruction of z-stacks and two-dimensional projections were conducted using the Imaris 7.1 Software (Bitplane Scientific Software) and the ZProjection ImageJ plugin, respectively. Mosaic images were taken using AxioVision software (Carl Zeiss). For confocal reflection microscopy, tumorospheres were seeded on glass bottom 35mm-μ-dishes and fixed with 4% formalin. Z-stack images were acquired using a Leica TCS-SP2 AOBS confocal microscopy with a HCX PL APO CS 40x/1.25 oil lens objective and a 488 nm Argon laser in reflection at room temperature.

### Statistical analysis

The two-tailed independent Student *t*-test was used to compare the variables between two groups. All data were derived from independent experiments. The level of statistical significance was set at 0.05 for all tests.

## SUPPLEMENTARY MATERIALS FIGURES AND VIDEOS














