# Supplementary material for: Control of long-distance cell-to-cell communication and autophagosome transfer in squamous cell carcinoma via tunneling nanotubes
Source: Oncotarget. 2017 Feb 18;8(13):20939–60. doi: 10.18632/oncotarget.15467 (PMC5400557; doi:10.18632/oncotarget.15467)
Supplement: Supplementary file 1 [file oncotarget-08-20939-s001.pdf]

Control of long-distance cell-to-cell communication and autophagosome transfer in squamous cell carcinoma via tunneling nanotubes

Supplementary Materials

A

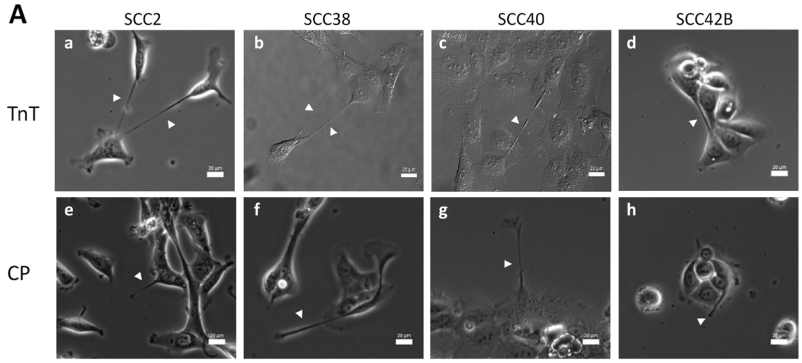

Cell line	Sex	Age (yrs)	Primary tumor location	TNM	Grade
SCC2	M	60	base of tongue	T4N1M0	G2
SCC38	M	66	glottic larynx	T2N0M0	G2
SCC40	M	65	tongue	T3N0M0	G1
SCC42B	M	43	supraglottic larynx	T4N3M0	G3

B

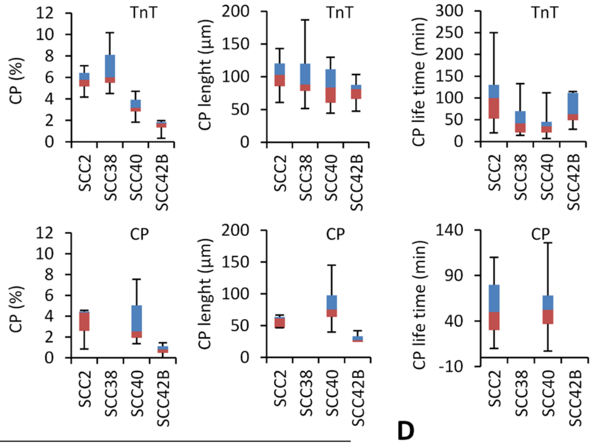

C

	minimum diameter of cell projections (µm)	
	TnT	CP
SCC2	1.47 ± 0.18	2.03 ± 0.07
SCC38	1.14 ± 0.47	
SCC40	1.35 ± 0.40	1.85 ± 0.63
SCC42B	1.16 ± 0.30	1.65 ± 0.96

D

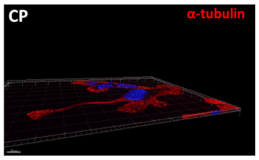

Supplementary Figure 1: Morphological properties of long cellular projections identified in SCC cell lines. (**A**) Phase (a, d-h) and differential interference contrast (b, c) images showing the two types of long cellular projections (TnTs and CPs, pointed by white arrows) found in the indicated SCC cell lines. Scale bars: 20 µm. The clinico-pathologic characteristics of the patients and tumor of origin of the SCC cell lines are indicated in the Table. M: male; yrs: years; TNM: tumor size (T), lymph nodes (N) and distant (M) metastasis; and differentiation grade (G1: well differentiated, G2: moderately differentiated and G3: poorly differentiated) are indicated in the table. (**B**) Quantification of the percentage of cells with TnTs or CP projections, and the maximal length and lifetime of the cell projections. Minimum diameter of the cell projections is described in Table (**C**). Data are presented as mean ± SD. *n* = 2 individual experiments and 1000 cells analyzed in each. (**D**) Three-dimensional reconstruction of Z-stacked images using Imaris software. Cells were inmunostained with the α-tubulin. The resulting image represent a stack of 15 sections (Z step of 3.92 µm) with a total physical length of 58.92 µm. Scale bar, 20 µm.

A

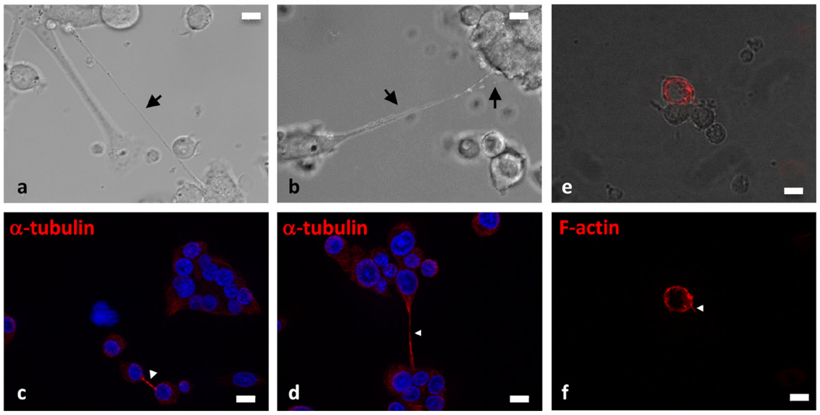

B

TnT PC12	
percentage	9.8
lenght (μm)	19±13
lifetime (min)	67±69
maximun diameter (μm)	1.23±0.61
minimun diameter (μm)	0.63±0.29

Supplementary Figure 2: TnTs in PC12 cells. (**A**) Phase contrast micrographs showing single (a) and branched TnTs (b) (indicated by black arrows). Image in b is a 57.6 μm Z-projection of 16 optical sections which allows visualizing a branched TnT. Micrographs c and d showing representative images of immunostaining for α-tubulin demonstrate that, in our culture conditions, TnTs of PC12 cells contain microtubules (indicated by white arrows). Representative phase contrast (e) and immunofluorescence (f) image of PC12 cells transiently transfected with LifeAct vector expressing a 17-amino acid peptide which stains filamentous F-actin structures. White arrow indicate a F-actin-stained TnT. (**B**) Table showing the mean and standard deviation of the indicated morphological features of TnTs found in PC12 cells (173 cells counted).

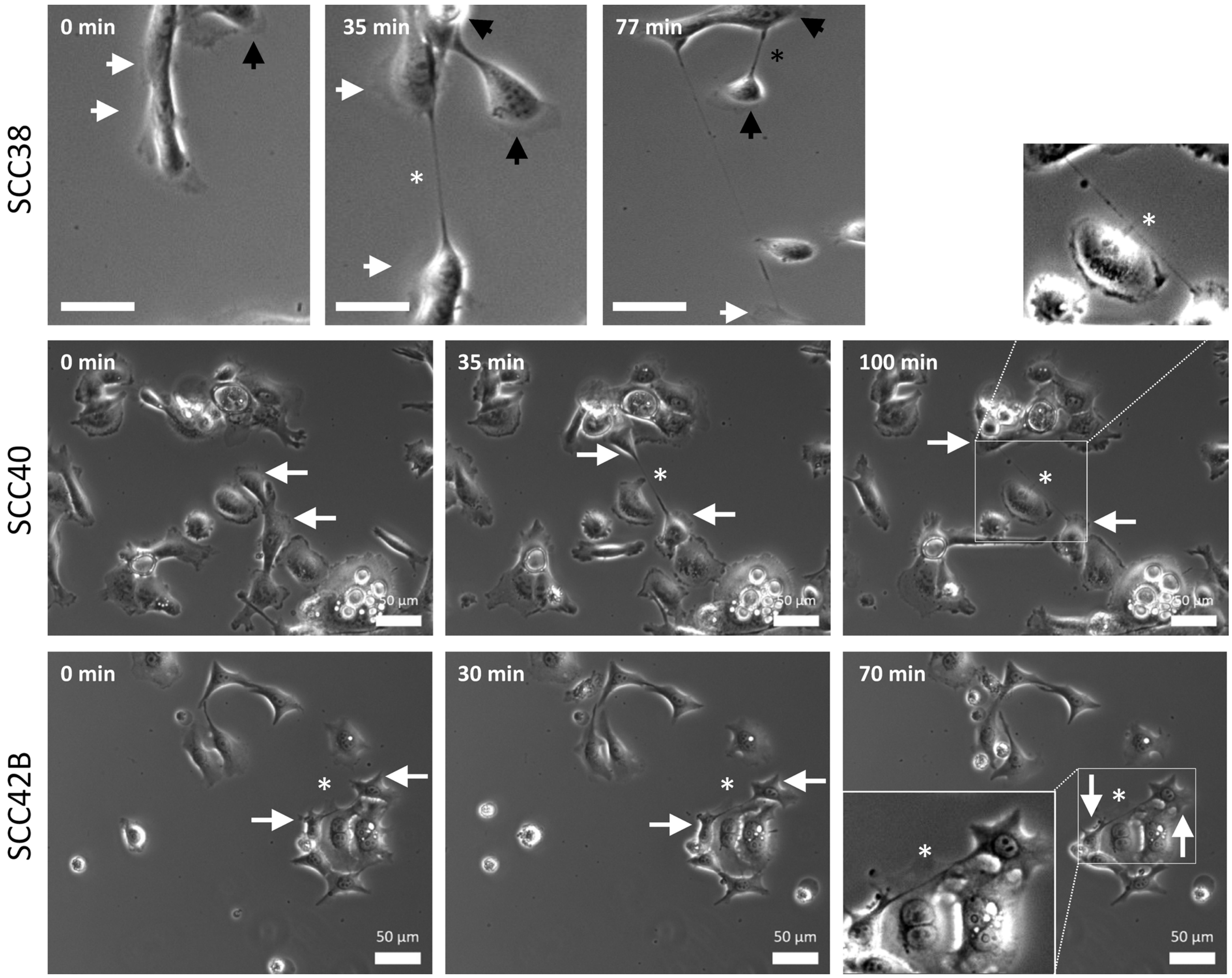

Supplementary Figure 3: SCC-TnTs are formed by cell-cell dislodgment (related to videos 2 and 3). Phase contrasts images of the indicated SCC cell lines at the indicated times. White and black arrows point to the cells involved in TnT formation. Asterisks denote TnTs. Scale bars, 50 µm.

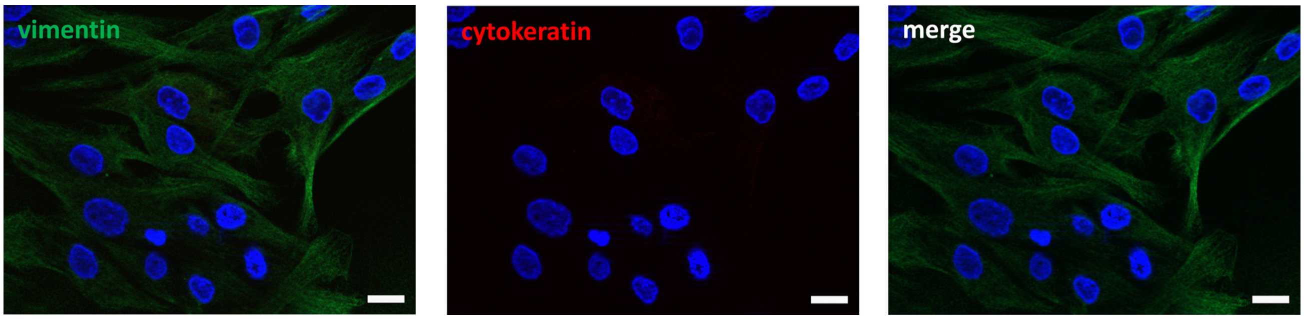

Supplementary Figure 4: Analysis of protein cell markers in CAFs derived from human SCC. Immunostaining with cytokeratin (**A**), or vimentin (**B**). Scale bar, 20 µm.

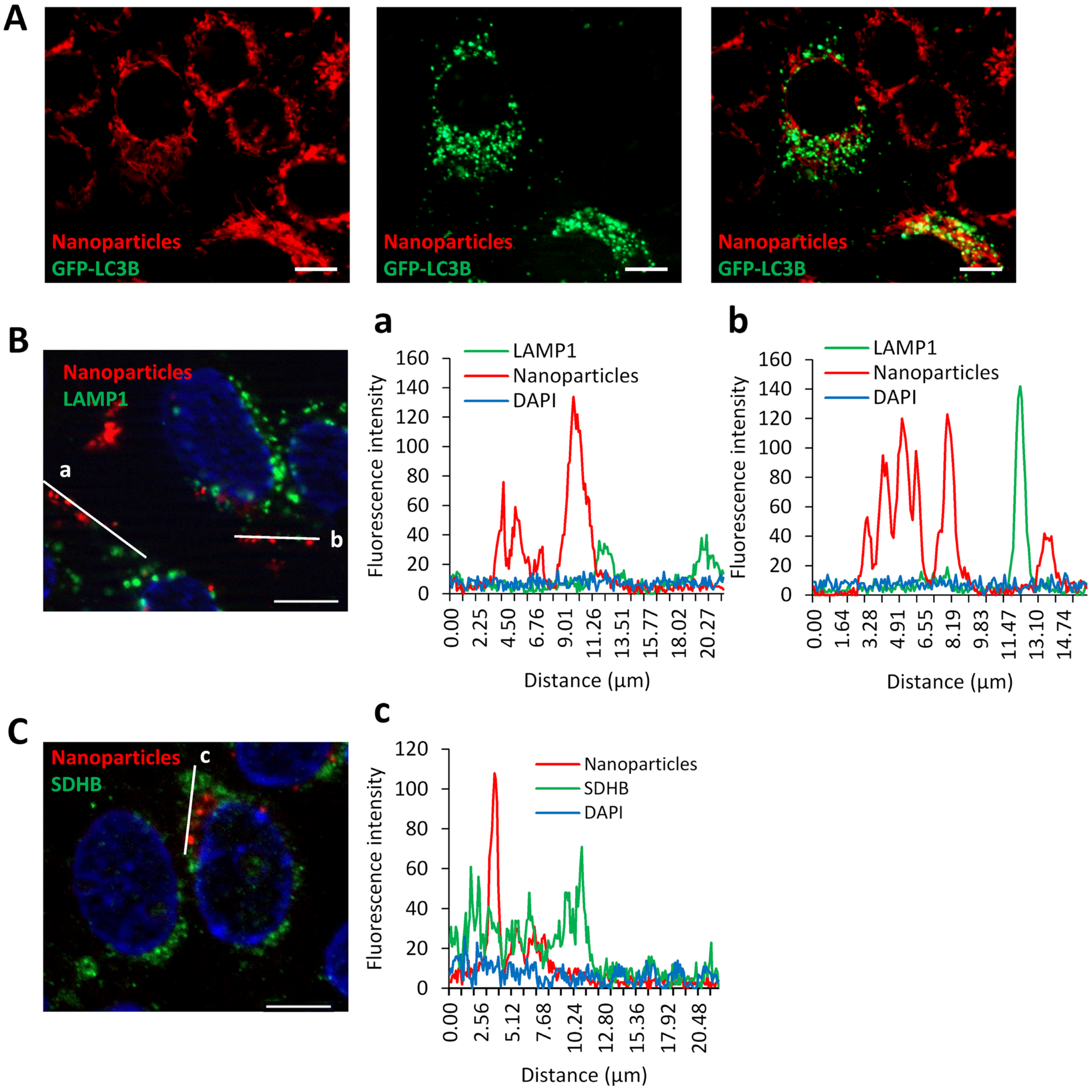

Supplementary Figure 5: Colocalization analysis of nanoparticles and lysosomes or mitochondria. (**A**) Representative fluorescence images of SCC38 cells transiently transfected with pMXs-IP-EGFP-LC3B and subsequently loaded with rhodamine-containing nanoparticles. (**B**, **C**) Immunofluorescence analysis of LAMP1 (B) and SDHB (C) in SCC38 cells loaded with rhodamine-containing nanoparticles. Graphics show the fluorescence intensity profiles of the ROIs (a-c) indicated in the pictures. Scale bars, 10 µm.

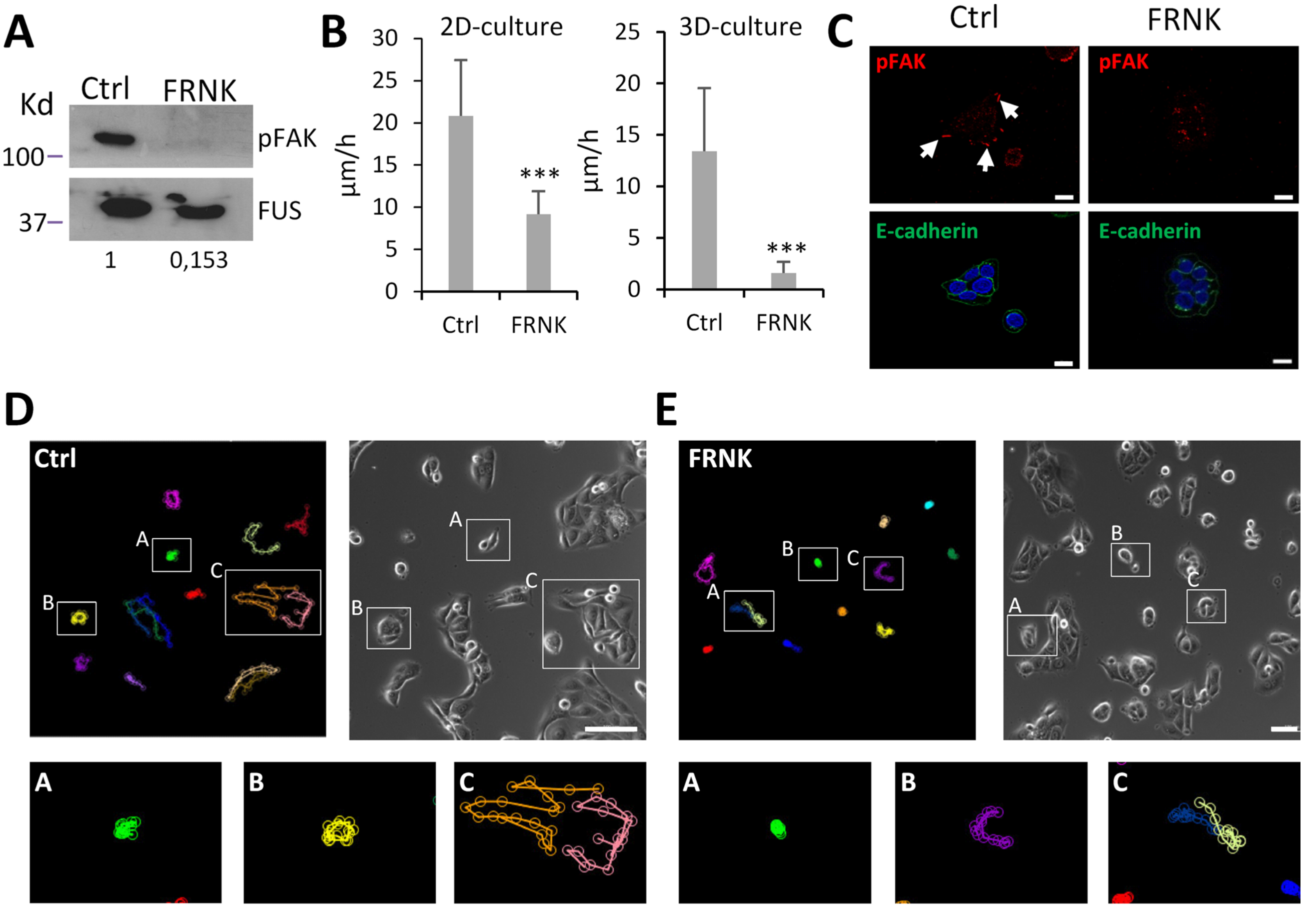

F Cell migration

	SCC38		SCC42B	
	μm/h	P	μm/h	P
Ctrl	78±27		21±6	
FRNK	48±11	0,001	9±2	1,34E-13
MMP-2	110±21	8,72E-05	15±3	0,015
FRNK-MMP-2	84±21	0,27	18±8	0,28

Supplementary Figure 6: Stably transfected pool populations of FRNK-SCC42B cells display reduced migration rate as compared with control cells. (**A**) Western blot analysis showing expression of pY395-FAK (pFAK) in SCC-42B cells stably transfected with pWZL- (Ctrl) or pWZL-FRNK (FRNK) vectors. Evaluation of Fused in Sarcoma (FUS) levels was used to assure even loading of proteins in each line. (**B**) Inhibition of FAK-mediated signaling by FRNK impairs single cell motility in SCC-42B cultured under two-dimensional (2D) or three-dimensional (3D, cells embedded in 2.5 mg/ml collagen matrix) conditions (2D culture: n = 1 experiment, 28 cells counted; 3D culture: n = 3 experiments, 529 cells counted). *** indicates $P < 0.0005$. (**C**) Representative images of staining for E-cadherin and pY397 FAK in SCC-42B cells showing that pFAK is localized to focal contacts in Ctrl-cells but it is absent in FRNK-cells. Scale bars, 20 μm. Ctrl- (**D**) and FRNK- (**E**) SCC42B cells were recorded by time-lapse video-microscopy for 19 hours and subsequently analyzed by computer-assisted cell-tracking. Representative images show that FRNK-SCC42B cells displayed reduced cell motility in contrast to Ctrl-SCC42B cells. Insets show magnified images indicated in the upper pictures. Scale bars, 100 μm. (**F**) Rates of cell migration of SCC42B cells (Ctrl, FRNK, MMP-2, and FRNK-MMP-2) as compared with SCC38 cells. As shown, MMP-2 over-expression in SCC cells restores the migratory ability of cells.

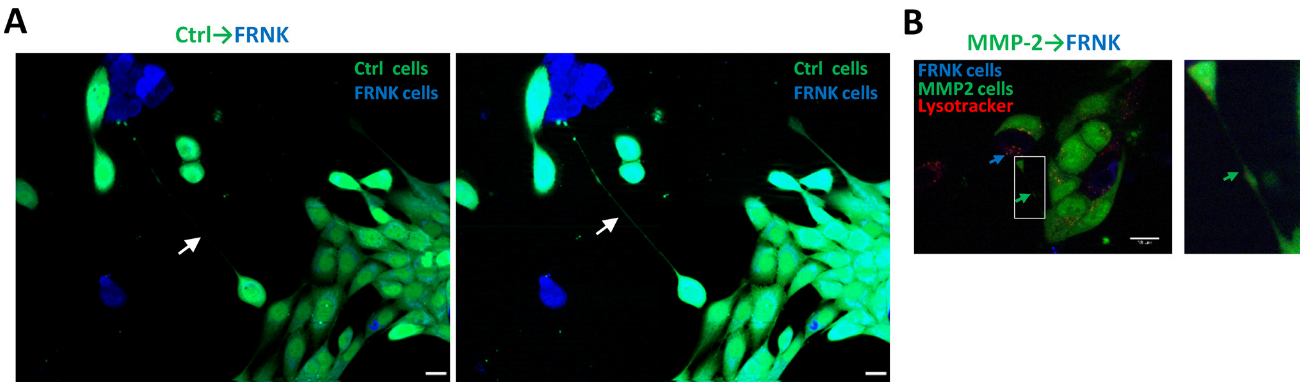

Supplementary Figure 7: TnT formation in co-culture experiments irrespective of the labeling procedure. FRNK-SCC38 cells labeled with blue CMAC dye were co-cultured with either Ctrl-SCC38 (**A**) or with MMP-2-SCC38 cells (**B**), both labeled with green CMFDA dye for 20 hours. Cells in B were also loaded with lysotracker. Micrograph at the right in A corresponds to an over-exposed image of the micrograph in the left to better visualize the TnT, denoted by a white arrow, that emanates from a FRNK cell and ends in a Ctrl cell. In B, a magnified image of the TnT framed in the left image is shown. The green arrow indicates a TnT that emanates from a MMP-2 cell and ends in a FRNK cell indicated by a blue arrow. Scale bars, 20 µm (A) and 16 µm (B).

Supplementary Video 1: TnTs formed in SCC38 cells. Linked to Figure 1E to show the mechanism of TnT. See_Supplementary_Video 1

Supplementary Video 2: TnTs formed in SCC40 cells. See_Supplementary_Video 2

Supplementary Video 3: TnTs formed in SCC42B cells. See_Supplementary_Video 3

Supplementary Video 4: Endosomal/Lysosomal vesicle transfer from cell to cell via TnT. SCC38 cells were labeled with green CMFDA and lysotracker 30 minutes before recording. Images were acquired every 5 minutes for 1 h. See_Supplementary_Video 4

Supplementary Video 5: Movement of mitochondria along TnT. See_Supplementary_Video 5

Supplementary Video 6: Motion of rhodamine-loaded polymeric nanoparticles along TnTs. SCC38 cells were incubated with rhodamine-loaded nanoparticles for 30 min before recording. Images were acquired every 2 min for 28 min. See_Supplementary_Video 6

SUPPLEMENTARY MATERIALS AND METHODS

Transient transfection and generation of stable FRNK-SCC42B and FRNK-MMP-2-SCC42B cell lines

Transient transfections of LifeAct plasmid (pLifeAct-tagRFP, ibidi) were performed using Lipofectamin 3000 Reagent (Invitrogen) according to the manufacturer's protocol. Stable populations of FRNK-SCC42B and FRNK-MMP-2-SCC42B cells were generated as previously described [2]. FRNK and MMP-2 expression in transfected pools were monitored by western blot analysis, real time PCR, and pFAK immunocytochemistry. For functional characterization, cell migration in collagen matrix was analyzed.

For western blot analyses, cells at 80% to 90% confluence were harvested by scraping on ice in lysis buffer containing 50 mmol/l HEPES (pH 7.9), 250 mmol/l NaCl, 5 mmol/l EDTA, 0.2% NP40, 10% glycerol, and protease inhibitors (0.5 mmol/l phenylmethylsulfonyl fluoride, 1 μg/ml aprotinin, 10 μg/ml leupeptin, 1mmol/l Na3VO4). Lysates were cleared by centrifugation (8000 × g) at 4°C for 5 minutes. The protein concentrations were determined in the supernatant with Bradford protein assay reagent (Bio-Rad). Equal amount of proteins (75 μg per lane) were resolved on 6% SDS-PAGE and transferred to Immun-Blot polyvinylidene difluoride membrane (Bio-Rad). Membranes were immunoblotted with mouse IgG anti-FAK (pY397) (BD Transduction Laboratories™) at 1:100 dilutions. For protein load control, mouse monoclonal anti-α-tubulin antibody (Sigma-Aldrich) was used at 1:5000 dilution. Immunodetection was done with the enhanced chemiluminescence plus Western blotting detection system (Millipore).

For quantitative real time RT-PCR, total RNA was isolated from cells as previously described (1). First-strand cDNA was synthesized from 2 μg of total RNA using the Superscript first-strand synthesis system for reverse transcriptase (Invitrogen, Carlsbad, CA, USA) with random primers according to the manufacturer’s directions. Real-time PCR was done in an ABI Prism 7500

Sequence Detection System (Applied Biosystems, Foster City, CA, USA) using SYBR Green PCR Master mix (Applied Biosystems) and the thermocycler conditions recommended by the manufacturer. Each sample was analyzed for cyclophilin A to normalize for RNA input amounts and to perform relative quantification. Primers were designed using the computer program Primer Express (Applied Biosys-tems). Primers were as follows: MMP-2, forward, 5′-TGCTGGAGACAAATTCTGGAGATA-3′; cyclophilin, forward, 5′-CATCTGCACTGCCAAGACTGA-3′ and reverse, 5′-TTGCCAAACACCACATGCTT-3′.

For culture of scattered cells in collagen matrix, 1 × 10^3 cells were mixed with collagen matrix, seeded onto a 60 μm-glass bottom dish, incubated for 30 min at 37°C, and then covered with growth medium. Phase contrast movies were taken at 10 min intervals for 24 h in a temperature, humidity, and CO_2 controlled environment. Movies were compiled and cell tracking was done using ImageJ MTrackJ and Manual Tracking Plug-In.

Generation of CAFs derived from surgically treated human SCC

CAFs were obtained from a surgically treated human SCC. Tumor tissues were enzymatically dissociated in PBS containing 1 mg/ml collagenase B for 1 h at 37°C with shaking and mechanic disruption every 10 min. After the enzymatic treatment, dissociated cells were centrifuged for 5 min at 200 × g and 4°C. Afterward, the pellet was resuspended in DMEN media supplemented with 10% fetal bovine serum, 1% penicillin/streptomycin, and 1% L-glutamine. Mild trypsinizations were subsequently conducted to recover the less adherent fibroblasts and get rid of any contaminating cancer cell. For immunocytochemistry studies, cells were seeded on culture slides the day before staining. CAFs expressed high levels of vimentin, a protein cell marker of mesenchymal cells, but not cytokeratin, a protein cell marker of epithelial cells (Supplementary Figure 4).

SCC tumor xenografts

Nude nu/nu mice were injected intraorally with either SCC38 or SCC42B cells as previously described [2]. Three weeks later, animals were sacrificed and tumors were analyzed by immunohistochemistry. The experiments were approved by the Ethics Committee for Medical Research of the University of Oviedo.

REFERENCES

1. Canel M, Secades P, Garzón-Arango M, Allonca E, Suarez C, Serrels A, Frame M, Brunton V, Chiara M-D. Involvement of focal adhesion kinase in cellular invasion of head and neck squamous cell carcinomas via regulation of MMP-2 expression. Br J Cancer. 2008; 98:1274–84.
2. Cabanillas R, Secades P, Rodrigo JP, Astudillo A, Suárez C, Chiara MD. [Orthotopic murine model of head and neck squamous cell carcinoma]. Acta Otorrinolaringol Esp. 2005; 56:89–95.
